# High-resolution HLA genetic diversity in a Northeastern Mexican clinical cohort

**DOI:** 10.3389/fgene.2026.1960786

**Published:** 2026-10-20

**Authors:** Jesús Antonio Ayala-Verdugo, José Carlos Jaime-Pérez, Sandra Iveth Mendoza-Ibarra, Carlos Octavio Cancela-Murrieta, Alondra Jetzany García-Valdez, Jorge Solis-Estrada, Rosario Salazar-Riojas, Andrés Gómez-De León, Perla Rocío Colunga-Pedraza, Olga Cantú-Rodríguez, David Gómez-Almaguer, Nidia Karina Moncada-Saucedo

**Affiliations:** Servicio de Hematología, Hospital Universitario “Dr. José Eleuterio González”, Universidad Autónoma de Nuevo León, Monterrey, Mexico

**Keywords:** Hardy–Weinberg equilibrium, HLA haplotypes, human leukocyte antigen (HLA), linkage disequilibrium, Mexican population, next-generation sequencing (NGS)

## Abstract

The human leukocyte antigen (HLA) region is the most polymorphic region of the human genome. Its allelic and haplotypic diversity varies markedly among populations, making it central to histocompatibility, transplantation, and population genetics. High-resolution data for northeastern Mexico remain scarce. We characterized HLA-A, -B, -C, and -DRB1 in a cohort of 606 unrelated individuals referred for histocompatibility testing between 2019 and 2024, using sequencing-based typing (SBT-Sanger) and next-generation sequencing (NGS) at two-field resolution. Allele and haplotype frequencies, Hardy–Weinberg equilibrium (HWE), the inbreeding coefficient (*F*_IS_), and linkage disequilibrium (LD) were analyzed using Arlequin v3.5.2.2 and R v4.5.2. HLA-A, -B, and -DRB1 conformed to HWE, whereas HLA-C showed a modest but significant deviation (*p* = 0.038). *F*_IS_ was close to zero at every locus (global *F*_IS_ = 0.00886), indicating no meaningful inbreeding. LD was significant for all locus pairs and strongest between HLA-B and HLA-C, with D′ approaching ±1 for many allelic combinations. HLA-A*03:01–B*07:02–C*07:02–DRB1*15:01 was one of the two most frequent haplotypes (1.24%). Pairwise fixation index (FST) analysis showed the lowest differentiation from admixed populations across the Americas and the greatest from Indigenous populations, most notably the Colombian North Chimila Amerindian population. These findings enhance the immunogenetic characterization of northeastern Mexico and provide a high-resolution regional reference for HLA diversity, with potential relevance to histocompatibility research.

## 1 Introduction

The human leukocyte antigen (HLA) region is the most polymorphic in the human genome. Its variation governs antigen presentation and shapes both transplant histocompatibility and susceptibility to immune-mediated disease, which is why HLA diversity matters as much in the clinic as in population genetics (Trowsdale and Knight, 2013; Meyer et al., 2018).

The distribution of HLA alleles and haplotypes differs substantially among populations as a result of evolutionary history and admixture. In Mexico, the genetic contribution of Indigenous American, European, and African ancestries has produced marked regional variation in HLA diversity. Although HLA variation has been described in several Mexican populations, high-resolution data remain scarce for many regions of the country, particularly northeastern Mexico. Broadening these datasets has a double payoff: it sharpens donor selection and histocompatibility assessment, and it gives population-structure and HLA–disease studies a regional baseline they currently lack (Barquera et al., 2020b).

Sequencing-based typing (SBT-Sanger) and next-generation sequencing (NGS) resolve HLA alleles at two-field resolution, well beyond what serology or low-resolution methods could achieve. This resolution also makes it possible to test Hardy–Weinberg equilibrium (HWE), estimate the inbreeding coefficient (*F*_IS_), and measure linkage disequilibrium (LD), metrics that describe how a population is structured and how internally consistent a high-polymorphism dataset is (Slatkin, 2008; Sanchez-Mazas et al., 2012; Montero-Martín et al., 2019).

In this study, we characterized high-resolution HLA-A, -B, -C, and -DRB1 allele and haplotype frequencies in a clinically referred cohort from northeastern Mexico. We further assessed its genetic structure through HWE, *F*_IS_, and LD analyses, and compared the cohort with reference populations across the Americas to contextualize its immunogenetic diversity. Our aim was to provide high-resolution regional immunogenetic reference data for histocompatibility research, future donor-search modeling, and transplantation studies in a region where such data remain limited.

## 2 Methods

### 2.1 Study population

This study included 606 unrelated individuals referred for clinical histocompatibility evaluation as potential donors for solid-organ or hematopoietic progenitor cell transplantation. Samples were received between January 2019 and December 2024 from our institution and collaborating hospitals and laboratories across northeastern Mexico. Only individuals with complete high-resolution HLA typing for HLA-A, HLA-B, HLA-C, and HLA-DRB1 were included in the analysis. Because participants were referred for histocompatibility rather than sampled from the general population, this is a clinically referred cohort rather than a population-based sample.

### 2.2 High-resolution HLA typing

Genomic DNA was extracted from peripheral blood using the EZ1 Advanced XL automated system with the EZ1 DNA Blood 350 µL Kit (QIAGEN, Hilden, Germany). High-resolution HLA typing of HLA-A, -B, -C, and -DRB1 was performed by SBT-Sanger for samples collected between January 2019 and January 2024 (468, 77.2%), using the AlleleSEQR HLA Sequencing Kit (GenDx, Utrecht, Netherlands), whereas samples collected between February and December 2024 (138, 22.8%) were typed by NGS using the AllType FASTplex NGS 11 Loci Flex Kit (One Lambda, Thermo Fisher Scientific, Canoga Park, CA, United States of America). Sequence analysis and allele assignment were performed using SBTengine v3.42 (GenDx) for Sanger and TypeStream Visual NGS v3.1 (One Lambda) for NGS.

Allele assignments were based on the IPD-IMGT/HLA database release available at the time of testing, ranging from versions 3.35–3.52 for SBT-Sanger and 3.52–3.57 for NGS, and were standardized to two-field resolution. When more than one compatible genotype remained at this resolution, the assignment used for analysis followed the prioritization provided by the corresponding software.

### 2.3 Population genetic analyses

Allele frequencies were estimated by direct counting. Extended haplotype frequencies were inferred using the expectation–maximization (EM) algorithm with bootstrap resampling implemented in Arlequin v3.5.2.2 (Excoffier and Lischer, 2010). HWE was evaluated using exact Markov chain Monte Carlo sampling, with significance set at *p* < 0.05 (Guo and Thompson, 1992). The *F*_IS_ was calculated following Wright’s (Wright, 1951) formulation from observed and expected heterozygosity. Pairwise LD between HLA loci was evaluated from genotypic data with unknown gametic phase using the likelihood-ratio test implemented in Arlequin v3.5.2.2 (Slatkin and Excoffier, 1996). Under this procedure, the likelihood of the observed genotypes under linkage equilibrium (LE) is compared with that obtained under linkage disequilibrium (LD), for which haplotype frequencies are estimated using the EM algorithm. The empirical null distribution of the likelihood-ratio statistic was generated using 10,100 permutations. D′ and *r* ^2^ were subsequently calculated in Arlequin from the EM-estimated haplotype frequencies to quantify the strength of allelic association. Population genetic analyses were performed using Arlequin v3.5.2.2, whereas R v4.5.2 was used for graphical visualization.

### 2.4 Population comparison

To place the northeastern Mexican cohort in a broader population context, pairwise genetic differentiation was evaluated against 22 comparison datasets derived from reference populations from North, Central, and South America retrieved from the Allele Frequency Net Database (AFND) (Gonzalez-Galarza et al., 2020). Only individuals with complete two-field HLA-A, HLA-B, HLA-C, and HLA-DRB1 genotypes were included from each dataset. Four-locus haplotype frequencies were inferred from the corresponding AFND genotype data using the EM algorithm implemented in Arlequin v3.5.2.2. Pairwise FST values were then estimated according to Weir and Cockerham (Weir and Cockerham, 1984), and statistical significance was assessed by 1,100 permutations. For composite datasets, individual-level genotypes from the corresponding AFND populations were combined before haplotype estimation and FST analysis (Supplementary Table S4).

### 2.5 Ethics statement

This retrospective study was approved by the Research and Ethics Committee of the University Hospital “Dr. José Eleuterio González”, Universidad Autónoma de Nuevo León (Approval No. HE25-00022). The study used anonymized data obtained during routine clinical HLA testing. The requirement for written informed consent was waived by the Ethics Committee due to the retrospective nature of the study.

## 3 Results

### 3.1 Allele frequency distribution

A total of 231 two-field alleles were identified across 52 HLA-A, 93 HLA-B, 38 HLA-C, and 48 HLA-DRB1. The most frequent HLA-A alleles were HLA-A*02:01 (23.76%), HLA-A*24:02 (14.44%), and HLA-A*03:01 (6.52%). The most frequent HLA-B alleles were HLA-B*35:01 (7.43%), HLA-B*40:02 (6.44%), and HLA-B*51:01 (6.11%). The most frequent HLA-C alleles were HLA-C*04:01 (17.16%), HLA-C*07:02 (14.77%), and HLA-C*07:01 (8.25%). For HLA-DRB1, the highest frequencies corresponded to HLA-DRB1*08:02 (11.88%), HLA-DRB1*04:07 (9.41%), and HLA-DRB1*07:01 (8.33%) (Table 1). The complete allele-frequency distribution is provided in Supplementary Table S2.

**TABLE 1:** Frequencies of the 20 most frequent HLA alleles by locus.

HLA-A allele	Freq. %	Cum. Freq. %	HLA-B allele	Freq. %	Cum. Freq. %	HLA-C allele	Freq. %	Cum. Freq. %	HLA-DRB1 allele	Freq. %	Cum. Freq. %
A*02:01	23.76	23.76	B*35:01	7.43	7.43	C*04:01	17.16	17.16	DRB1*08:02	11.88	11.88
A*24:02	14.44	38.20	B*40:02	6.44	13.87	C*07:02	14.77	31.93	DRB1*04:07	9.41	21.29
A*03:01	6.52	44.72	B*51:01	6.11	19.98	C*07:01	8.25	40.18	DRB1*07:01	8.33	29.62
A*31:01	5.45	50.17	B*07:02	4.54	24.51	C*03:04	7.43	47.61	DRB1*04:04	6.68	36.31
A*02:06	5.20	55.37	B*39:05	4.21	28.72	C*01:02	6.93	54.54	DRB1*03:01	6.19	42.50
A*01:01	4.87	60.23	B*44:03	4.13	32.85	C*06:02	4.62	59.16	DRB1*15:01	5.86	48.35
A*11:01	4.87	65.10	B*14:02	3.80	36.64	C*08:02	4.37	63.53	DRB1*13:01	4.29	52.65
A*68:01	4.37	69.47	B*49:01	3.63	40.27	C*16:01	4.13	67.66	DRB1*14:06	4.21	56.85
A*68:03	2.89	72.36	B*39:06	3.47	43.74	C*12:03	4.13	71.79	DRB1*13:02	3.14	59.99
A*29:02	2.72	75.08	B*18:01	3.38	47.12	C*03:03	3.80	75.58	DRB1*01:01	3.05	63.04
A*23:01	2.56	77.64	B*52:01	3.05	50.17	C*08:01	3.30	78.88	DRB1*16:02	2.97	66.01
A*33:01	2.56	80.20	B*35:12	2.97	53.14	C*05:01	3.14	82.02	DRB1*11:04	2.97	68.98
A*32:01	2.48	82.68	B*48:01	2.81	55.95	C*02:02	2.72	84.74	DRB1*04:03	2.89	71.87
A*26:01	2.31	84.99	B*08:01	2.64	58.59	C*15:02	2.48	87.21	DRB1*04:11	2.72	74.59
A*30:02	2.31	87.30	B*15:01	2.48	61.06	C*03:06	1.90	89.11	DRB1*11:01	2.64	77.23
A*68:02	1.82	89.11	B*44:02	1.98	63.04	C*17:01	1.57	90.68	DRB1*14:02	2.56	79.79
A*02:05	1.65	90.76	B*35:03	1.82	64.86	C*12:02	1.32	92.00	DRB1*01:02	2.39	82.18
A*68:05	1.57	92.33	B*35:17	1.82	66.68	C*14:02	1.24	93.24	DRB1*04:05	1.65	83.84
A*30:01	1.40	93.73	B*40:05	1.82	68.49	C*02:10	1.16	94.39	DRB1*14:54	1.40	85.24
A*25:01	1.07	94.80	B*15:15	1.73	70.22	C*03:05	1.07	95.46	DRB1*15:02	1.16	86.39

*Freq.: Frequency; Cum. Freq.: Cumulated frequency*.

### 3.2 Extended haplotype diversity

A large number of four-locus haplotypes were inferred, although only four exceeded a frequency of 1%. The most frequent haplotypes, each at 1.24%, were HLA-A*03:01∼HLA-B*07:02∼HLA-C*07:02∼HLA-DRB1*15:01 and HLA-A*68:03∼HLA-B*39:05∼HLA-C*07:02∼HLA-DRB1*04:07. Two additional haplotypes exceeded 1%, at 1.16% (A*24:02∼B*39:06∼C*07:02∼DRB1*14:06) and 1.07% (A*29:02∼B*44:03∼C*16:01∼DRB1*07:01). The remaining haplotypes were all observed at frequencies below 1%, indicating extensive haplotypic diversity within the cohort (Table 2). The complete distribution of inferred four-locus haplotypes is provided in Supplementary Table S3.

**TABLE 2:** Distribution of the 20 most frequent HLA haplotypes.

Haplotype	Freq. %	Cum. Freq. %	Haplotype	Freq. %	Cum. Freq. %
A*03:01∼B*07:02∼C*07:02∼DRB1*15:01	1.24	1.24	A*02:01∼B*44:03∼C*04:01∼DRB1*07:01	0.58	10.40
A*68:03∼B*39:05∼C*07:02∼DRB1*04:07	1.24	2.48	A*24:02∼B*40:05∼C*03:04∼DRB1*04:04	0.58	10.97
A*24:02∼B*39:06∼C*07:02∼DRB1*14:06	1.16	3.63	A*30:02∼B*18:01∼C*05:01∼DRB1*03:01	0.58	11.55
A*29:02∼B*44:03∼C*16:01∼DRB1*07:01	1.07	4.70	A*02:06∼B*39:05∼C*07:02∼DRB1*04:07	0.58	12.13
A*02:01∼B*35:17∼C*04:01∼DRB1*08:02	0.99	5.69	A*24:02∼B*35:12∼C*04:01∼DRB1*08:02	0.50	12.62
A*01:01∼B*08:01∼C*07:01∼DRB1*03:01	0.91	6.60	A*24:02∼B*40:02∼C*03:06∼DRB1*08:02	0.50	13.12
A*02:01∼B*35:12∼C*04:01∼DRB1*08:02	0.83	7.43	A*02:01∼B*35:12∼C*04:01∼DRB1*04:07	0.50	13.61
A*03:01∼B*51:01∼C*16:01∼DRB1*07:01	0.83	8.25	A*02:01∼B*50:01∼C*06:02∼DRB1*03:01	0.50	14.11
A*33:01∼B*14:02∼C*08:02∼DRB1*01:02	0.83	9.08	A*24:02∼B*40:05∼C*03:04∼DRB1*08:02	0.50	14.60
A*24:02∼B*35:01∼C*04:01∼DRB1*04:04	0.74	9.82	A*02:01∼B*35:01∼C*04:01∼DRB1*08:02	0.50	15.10

*Freq.: Frequency; Cum. Freq.: Cumulated frequency*.

### 3.3 Hardy–Weinberg equilibrium and inbreeding coefficient

Three of the four loci were in HWE. No significant departures were detected for HLA-A (Ho = 0.89934; He = 0.90111; *p* = 0.40519), HLA-B (Ho = 0.95875; He = 0.96895; *p* = 0.64448), or HLA-DRB1 (Ho = 0.92079; He = 0.94698; *p* = 0.27685). HLA-C showed a modest but statistically significant departure from HWE, characterized by a slight excess of heterozygotes (Ho = 0.92409; He = 0.91903; *p* = 0.03804) (Supplementary Table S1).

*F*_IS_ values were close to zero at all loci. Positive values were observed for HLA-A (0.00196), HLA-B (0.01053), and HLA-DRB1 (0.02768), whereas HLA-C showed a slightly negative *F*_IS_ (−0.00551), consistent with the modest heterozygote excess observed at this locus. The heterozygosity-weighted global *F*_IS_ was 0.00886.

### 3.4 Linkage disequilibrium

Significant LD was detected for all six pairwise comparisons among the four HLA loci using the likelihood-ratio permutation test for genotypic data with unknown gametic phase (10,100 permutations; empirical *p* < 0.0001 for all comparisons). The strongest LD was observed between HLA-B and HLA-C, whereas the remaining locus pairs also showed significant but less pronounced LD. Pairwise D′ and *r* ^2^ estimates for the 20 most frequent alleles at each locus are presented in Figure 1.

**FIGURE 1:**
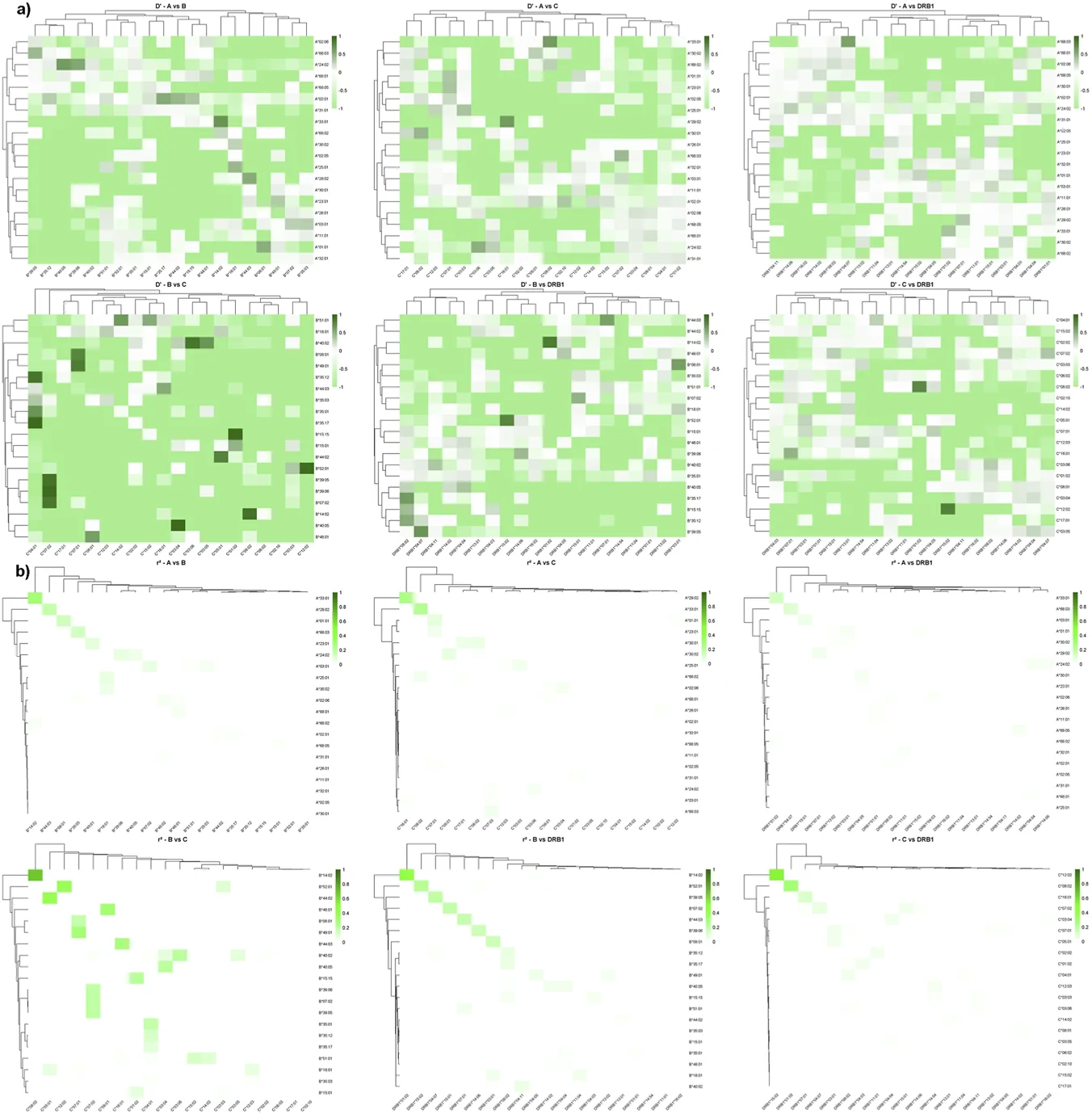
Linkage disequilibrium patterns among the 20 most frequent HLA-A, -B, -C, and -DRB1 alleles in the northeastern Mexican cohort. Heatmaps illustrate pairwise linkage disequilibrium for the six locus combinations (HLA-A/HLA-B, HLA-A/HLA-C, HLA-A/HLA-DRB1, HLA-B/HLA-C, HLA-B/HLA-DRB1, and HLA-C/HLA-DRB1). **(a)** Standardized linkage disequilibrium (D′) values. Values approaching ±1 indicate little or no historical recombination, whereas values near 0 are consistent with linkage equilibrium. **(b)** Squared correlation coefficient (r^2^) values. Higher r^2^ values indicate stronger allelic correlation.

### 3.5 Genetic differentiation among American populations

Pairwise FST analysis placed the northeastern Mexican cohort within the genetic landscape of the Americas (Figure 2). The lowest levels of genetic differentiation were observed in admixed populations, including the Brazilian, Central American Mestizo, Colombian cord blood, and San Diego cohorts, all with FST values below 0.003, consistent with high haplotypic similarity to our cohort. In contrast, Indigenous populations, including Maya, Mixe/Mixtec, Ojibwa, Chipewyan, Aymara/Quechua, and Puyanawa datasets; and Colombian Amerindian groups (COL NC-Ame and COL N-EE), displayed higher genetic differentiation (FST = 0.0045–0.0494). The greatest differentiation was observed for the Colombian North Chimila Amerindian population (COL NC-Ame; FST = 0.0494), followed by the Colombian North Wiwa population (COL N-EE; FST = 0.0246). All pairwise comparisons with the northeastern Mexican cohort were statistically significant (*p* < 0.05).

**FIGURE 2:**
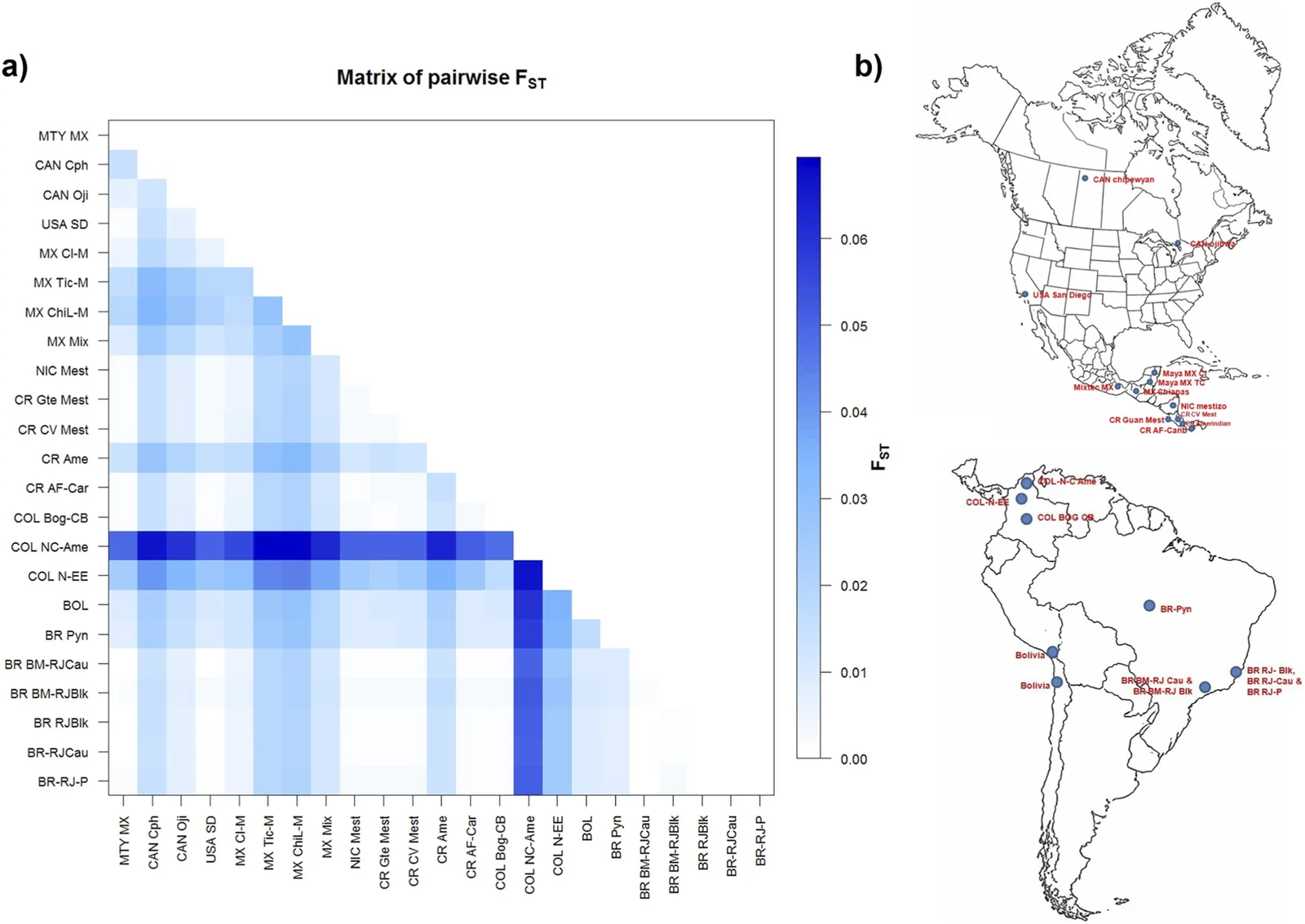
Pairwise genetic differentiation between the northeastern Mexican cohort and reference populations from the Americas. **(a)** Heatmap showing pairwise FST values calculated from four-locus HLA haplotypes (HLA-A ∼ B ∼ C ∼ DRB1). Darker shades indicate greater genetic differentiation between populations. **(b)** Geographic origin of the reference populations included in the pairwise FST analysis. Population abbreviations: CAN Cph, Canadian Chipewyan; CAN Oji, Canadian Ojibwa; USA SD, United States of America San Diego; MX CI-M, Chichén Itzá Maya; MX Tic-M, Tixcacaltuyub Maya; MX ChiL-M, Lacandon Maya; MX Mix, Mexico Mixe + Mixtec composite dataset; NIC Mest, Nicaragua Mestizo; CR Gte Mest, Guanacaste Mestizo; CR CV Mest, Central Valley Mestizo; CR Ame, Costa Rica Amerindian; CR AF-Car, Costa Rica African-Caribbean; COL Bog-CB, Bogotá Cord Blood; COL NC-Ame, North Chimila Amerindian; COL N-EE, North Wiwa (El Encanto); BOL, Bolivia/Chile Aymara + Bolivia/Peru Quechua composite dataset; BR Pyn, Puyanawa; BR BM-RJCau, Barra Mansa Caucasian; BR BM-RJBlk, Barra Mansa Black; BR RJBlk, Rio de Janeiro Black; BR-RJCau, Rio de Janeiro Caucasian; BR-RJ-P, Rio de Janeiro Parda.

## 4 Discussion

The present study provides a high-resolution characterization of HLA diversity in a clinically referred cohort from northeastern Mexico. The data generated expand the immunogenetic characterization of this region and provide a regional reference for histocompatibility studies performed in routine clinical practice.

The allele and haplotype distributions match those reported for admixed Mexican populations, in which a few common alleles account for much of the diversity while many low-frequency alleles sustain the overall polymorphism (Barquera et al., 2020b; 2020a). Several of the recurrent four-locus haplotypes have been described in earlier Mexican and Latin American series (Cortes et al., 2004; Barquera et al., 2008; Mancías-Guerra et al., 2024), supporting the regional relevance of the haplotype structure observed in our cohort, although referral- or sampling-related effects cannot be excluded.

Of the four loci analyzed, only HLA-C departed from HWE, showing a modest but statistically significant departure from Hardy–Weinberg equilibrium, characterized by a slight excess of heterozygotes (*p* = 0.038). HLA loci are known to be shaped by evolutionary forces such as balancing selection and pathogen-mediated selective pressures, which contribute to the maintenance of extensive polymorphism across the MHC (Meyer and Thomson, 2001; Meyer et al., 2018). However, the present study was designed to characterize regional HLA diversity rather than to test selective mechanisms, and the observed HLA-C deviation cannot be attributed to selection on the basis of these data alone.

Population structure related to the admixture history of northeastern Mexico may also contribute to the observed pattern, although its effect on HWE depends on the underlying population structure (Sanchez-Mazas et al., 2012; Barquera et al., 2020b). In addition, the interpretation of HWE testing at highly polymorphic HLA loci may be influenced by sample size relative to allelic diversity and by sparse genotype counts associated with rare alleles (Guo and Thompson, 1992; Mack et al., 2012). These factors, together with the clinically referred nature of the cohort, should therefore be considered alongside possible selective pressures when interpreting the modest HLA-C deviation. The small differences between observed and expected heterozygosity at HLA-A, HLA-B, and HLA-DRB1, together with the near-zero global *F*_IS_ (0.00886), are consistent with limited overall heterozygosity deviation in the cohort.

HLA-C also has clinical relevance in histocompatibility assessment. High-resolution HLA-C matching forms part of contemporary unrelated-donor selection strategies, and HLA-C mismatching has been associated with adverse outcomes after hematopoietic cell transplantation (Woolfrey et al., 2011; Jimenez Jimenez et al., 2025). In the context of the present study, these observations support the value of characterizing regional HLA-C diversity at high resolution; however, transplantation outcomes and the effects of specific HLA-C mismatches were not evaluated in this cohort.

The strongest LD involved HLA-B and HLA-C, in line with their physical proximity and the low recombination of this segment of the MHC (Trowsdale and Knight, 2013). The recurrent extended haplotypes identified in this cohort reinforce that conserved chromosomal blocks persist across the region (Slatkin, 2008; Shao et al., 2016).

The D′ and *r* ^2^ estimates separate two things that are easy to conflate: high D′ across many allele pairs points to limited historical recombination, whereas the wide spread of *r* ^2^ reflects that predictability between alleles is capped by their frequencies. Strong LD therefore does not guarantee high predictive power—*r* ^2^ is pulled down when allele frequencies are mismatched, as happens with low-frequency alleles even when D′ approaches 1 (VanLiere and Rosenberg, 2008).

Against reference populations of the Americas, the cohort was almost indistinguishable from other admixed populations (FST< 0.003) and progressively more differentiated from Indigenous/Amerindian groups (FST = 0.0045–0.0494), the largest being the North Chimila Amerindians of Colombia (FST = 0.0494). Notably, the cohort clustered closer to admixed populations from other countries than to the Indigenous populations of Mexico itself, consistent with the high European component reported for northern Mexico and with the population-specific haplotypes that Indigenous groups retain through drift and selection (Barquera et al., 2008; 2020b; Meyer et al., 2018).

Previous HLA data from northeastern Mexico are scarce and have been largely limited to low-resolution typing or small high-resolution cohorts. Earlier studies from Nuevo León characterized HLA diversity using PCR-SSP (Barquera et al., 2020a; Mancías-Guerra et al., 2022), whereas our previous high-resolution data were limited to a preliminary report on a pilot transplant cohort (Mancías-Guerra et al., 2024). By analyzing 606 unrelated individuals at two-field resolution, the present study substantially expands these observations and provides the most detailed high-resolution HLA characterization reported for northeastern Mexico to date.

The practical relevance of these data lies in providing regional estimates of HLA allele and extended haplotype frequencies for northeastern Mexico. Such information does not replace individual high-resolution HLA typing or established donor-selection criteria but provides a population framework in which donor searches may be interpreted. Population-specific high-resolution haplotype frequencies are an important component of models used to estimate the likelihood of identifying an HLA-matched unrelated donor, and substantial differences in match likelihood have been demonstrated among populations with distinct HLA frequency distributions (Gragert et al., 2014). The extensive haplotypic diversity observed in our cohort, with only four haplotypes exceeding a frequency of 1%, further highlights the value of region-specific HLA frequency data. Knowledge of which alleles and haplotypes are common or comparatively uncommon may help contextualize donor-search expectations and, together with larger population-based datasets and registry information, may inform future donor-search modeling and studies of regional HLA representation.

A few methodological and sampling aspects should be considered when interpreting these findings. The cohort included 606 unrelated individuals referred for histocompatibility testing and may therefore not fully represent the broader northeastern Mexican population. Typing was performed by SBT-Sanger and NGS, with allele assignments harmonized at two-field resolution. Given the high polymorphism of the HLA system, some rare alleles and haplotypes were represented by small counts. Four-locus haplotypes were statistically inferred, and the analysis focused on HLA-A, -B, -C, and -DRB1. Future population-based studies with broader sampling and additional HLA loci may further complement this regional reference.

This study provides a high-resolution characterization of HLA-A, -B, -C, and -DRB1 diversity in a clinically referred cohort from northeastern Mexico, a region for which two-field HLA data remain limited. Because participants were referred for routine histocompatibility testing rather than recruited through population-based sampling, the allele and haplotype frequencies reported here should not be interpreted as definitive estimates for the entire northeastern Mexican population. Nevertheless, their concordance with previously reported Mexican HLA patterns and the low genetic differentiation observed with several admixed populations across the Americas support the regional relevance of the dataset. Future population-based studies incorporating broader geographic sampling and additional HLA loci will be needed to further define the immunogenetic diversity of northeastern Mexico. Overall, this study expands the high-resolution HLA information available for an underrepresented region and provides a foundation for future research in histocompatibility, transplantation, population genetics, and human immunogenetics.

## Data Availability

The original contributions presented in the study are included in the article/Supplementary Material, further inquiries can be directed to the corresponding author.
