# Supplementary material for: High-resolution HLA genetic diversity in a Northeastern Mexican clinical cohort
Source: Front Genet. 2026 Oct 20;17:1960786. doi: 10.3389/fgene.2026.1960786 (PMC13727317; doi:10.3389/fgene.2026.1960786)
Supplement: Supplementary file 1 [file Table1.docx]

Supplementary Material

**Supplementary Table S1.** Summary of Hardy–Weinberg equilibrium statistics and heterozygosity estimates for HLA-A, HLA-B, HLA-C, and HLA-DRB1.

| **Locus** | **H_o_** | **H_e_** | **HWE**  **p-value** | **FIS** | | **Interpretation** | **Weighted FIS contribution**  **(FIS × H_e_)** |
| --- | --- | --- | --- | --- | --- | --- | --- |
| **HLA-A** | 0.89934 | 0.90111 | 0.40519 | | 0.00196 | Minimal heterozygote deficit | 0.001766 |
| **HLA-B** | 0.95875 | 0.96895 | 0.64448 | | 0.01053 | Slight heterozygote deficit | 0.010202 |
| **HLA-C** | 0.92409 | 0.91903 | 0.03804 | | -0.00551 | Heterozygote excess | -0.005064 |
| **HLA-DRB1** | 0.92079 | 0.94698 | 0.27685 | | 0.02768 | Moderate heterozygote deficit | 0.026183 |
| *Abbreviations:* HWE, Hardy–Weinberg equilibrium; Ho, observed heterozygosity; He, expected heterozygosity; FIS, inbreeding coefficient. The overall FIS estimated across all loci was 0.00886. | | | | | | | |

**Supplementary Table S2.** Complete two-field HLA-A, -B, -C, and -DRB1 allele frequency distribution in the clinically referred cohort from northeastern Mexico.

| HLA-A | Frequency  % | HLA-B | Frequency  % | HLA-C | Frequency  % | HLA-DRB1 | Frequency  % |
| --- | --- | --- | --- | --- | --- | --- | --- |
| A*02:01 | 23.76 | **B*35:01** | 7.43 | **C*04:01** | 17.16 | **DRB1*08:02** | 11.88 |
| A*24:02 | 14.44 | **B*40:02** | 6.44 | **C*07:02** | 14.77 | **DRB1*04:07** | 9.41 |
| A*03:01 | 6.52 | **B*51:01** | 6.11 | **C*07:01** | 8.25 | **DRB1*07:01** | 8.33 |
| A*31:01 | 5.45 | **B*07:02** | 4.54 | **C*03:04** | 7.43 | **DRB1*04:04** | 6.68 |
| A*02:06 | 5.20 | **B*39:05** | 4.21 | **C*01:02** | 6.93 | **DRB1*03:01** | 6.19 |
| A*01:01 | 4.87 | **B*44:03** | 4.13 | **C*06:02** | 4.62 | **DRB1*15:01** | 5.86 |
| A*11:01 | 4.87 | **B*14:02** | 3.80 | **C*08:02** | 4.37 | **DRB1*13:01** | 4.29 |
| A*68:01 | 4.37 | **B*49:01** | 3.63 | **C*16:01** | 4.13 | **DRB1*14:06** | 4.21 |
| A*68:03 | 2.89 | **B*39:06** | 3.47 | **C*12:03** | 4.13 | **DRB1*13:02** | 3.14 |
| A*29:02 | 2.72 | **B*18:01** | 3.38 | **C*03:03** | 3.80 | **DRB1*01:01** | 3.05 |
| A*23:01 | 2.56 | **B*52:01** | 3.05 | **C*08:01** | 3.30 | **DRB1*16:02** | 2.97 |
| A*33:01 | 2.56 | **B*35:12** | 2.97 | **C*05:01** | 3.14 | **DRB1*11:04** | 2.97 |
| A*32:01 | 2.48 | **B*48:01** | 2.81 | **C*02:02** | 2.72 | **DRB1*04:03** | 2.89 |
| A*26:01 | 2.31 | **B*08:01** | 2.64 | **C*15:02** | 2.48 | **DRB1*04:11** | 2.72 |
| A*30:02 | 2.31 | **B*15:01** | 2.48 | **C*03:06** | 1.90 | **DRB1*11:01** | 2.64 |
| A*68:02 | 1.82 | **B*44:02** | 1.98 | **C*17:01** | 1.57 | **DRB1*14:02** | 2.56 |
| A*02:05 | 1.65 | **B*35:03** | 1.82 | **C*12:02** | 1.32 | **DRB1*01:02** | 2.39 |
| A*68:05 | 1.57 | **B*35:17** | 1.82 | **C*14:02** | 1.24 | **DRB1*04:05** | 1.65 |
| A*30:01 | 1.40 | **B*40:05** | 1.82 | **C*02:10** | 1.16 | **DRB1*14:54** | 1.40 |
| A*25:01 | 1.07 | **B*15:15** | 1.73 | **C*03:05** | 1.07 | **DRB1*15:02** | 1.16 |
| A*66:01 | 0.41 | **B*27:05** | 1.73 | **C*15:09** | 0.74 | **DRB1*08:01** | 1.07 |
| A*01:02 | 0.33 | **B*38:01** | 1.65 | **C*08:03** | 0.58 | **DRB1*08:04** | 1.07 |
| A*02:02 | 0.33 | **B*50:01** | 1.57 | **C*16:02** | 0.58 | **DRB1*09:01** | 1.07 |
| A*24:07 | 0.33 | **B*15:03** | 1.32 | **C*03:02** | 0.41 | **DRB1*03:02** | 0.99 |
| A*24:25 | 0.33 | **B*15:30** | 1.24 | **C*07:04** | 0.41 | **DRB1*16:01** | 0.91 |
| A*74:01 | 0.33 | **B*55:01** | 1.24 | **C*15:05** | 0.25 | **DRB1*04:02** | 0.83 |
| A*02:20 | 0.25 | **B*53:01** | 1.16 | **C*06:01** | 0.25 | **DRB1*01:03** | 0.74 |
| A*24:03 | 0.25 | **B*35:08** | 1.07 | **C*17:03** | 0.25 | **DRB1*10:01** | 0.74 |
| A*29:01 | 0.25 | **B*41:01** | 1.07 | **C*03:08** | 0.17 | **DRB1*04:01** | 0.66 |
| A*34:02 | 0.25 | **B*35:02** | 0.91 | **C*04:04** | 0.17 | **DRB1*11:02** | 0.66 |
| A*69:01 | 0.25 | **B*39:02** | 0.91 | **C*07:18** | 0.17 | **DRB1*15:03** | 0.66 |
| A*31:02 | 0.17 | **B*57:01** | 0.91 | **C*04:82** | 0.08 | **DRB1*13:04** | 0.50 |
| A*68:07 | 0.17 | **B*13:02** | 0.83 | **C*07:17** | 0.08 | **DRB1*13:05** | 0.50 |
| A*01:17 | 0.08 | **B*14:01** | 0.83 | **C*07:35** | 0.08 | **DRB1*04:10** | 0.41 |
| A*02:09 | 0.08 | **B*39:01** | 0.83 | **C*08:32** | 0.08 | **DRB1*04:08** | 0.33 |
| A*02:11 | 0.08 | **B*51:02** | 0.83 | **C*12:04** | 0.08 | **DRB1*12:02** | 0.33 |
| A*02:22 | 0.08 | **B*35:43** | 0.74 | **C*16:04** | 0.08 | **DRB1*13:03** | 0.33 |
| A*02:35 | 0.08 | **B*40:08** | 0.74 | **C*18:01** | 0.08 | **DRB1*14:01** | 0.33 |
| A*03:02 | 0.08 | **B*58:01** | 0.66 |  |  | **DRB1*08:03** | 0.25 |
| A*24:01 | 0.08 | **B*37:01** | 0.58 |  |  | **DRB1*11:03** | 0.25 |
| A*24:15 | 0.08 | **B*39:08** | 0.58 |  |  | **DRB1*14:07** | 0.25 |
| A*24:23 | 0.08 | **B*42:01** | 0.50 |  |  | **DRB1*12:01** | 0.17 |
| A*24:33 | 0.08 | **B*41:02** | 0.41 |  |  | **DRB1*14:24** | 0.17 |
| A*26:03 | 0.08 | **B*42:02** | 0.41 |  |  | **DRB1*04:25** | 0.08 |
| A*30:04 | 0.08 | **B*45:01** | 0.41 |  |  | **DRB1*08:06** | 0.08 |
| A*31:09 | 0.08 | **B*15:17** | 0.33 |  |  | **DRB1*08:07** | 0.08 |
| A*33:03 | 0.08 | **B*35:14** | 0.33 |  |  | **DRB1*09:06** | 0.08 |
| A*36:01 | 0.08 | **B*35:16** | 0.33 |  |  | **DRB1*13:15** | 0.08 |
| A*68:12 | 0.08 | **B*35:28** | 0.33 |  |  |  |  |
| A*68:15 | 0.08 | **B*40:01** | 0.33 |  |  |  |  |
| A*68:20 | 0.08 | **B*44:05** | 0.33 |  |  |  |  |
| A*80:01 | 0.08 | **B*56:01** | 0.33 |  |  |  |  |
|  |  | **B*16:01** | 0.25 |  |  |  |  |
|  |  | **B*40:06** | 0.25 |  |  |  |  |
|  |  | **B*40:27** | 0.17 |  |  |  |  |
|  |  | **B*50:02** | 0.17 |  |  |  |  |
|  |  | **B*15:10** | 0.17 |  |  |  |  |
|  |  | **B*15:35** | 0.17 |  |  |  |  |
|  |  | **B*27:02** | 0.17 |  |  |  |  |
|  |  | **B*35:04** | 0.17 |  |  |  |  |
|  |  | **B*35:20** | 0.17 |  |  |  |  |
|  |  | **B*40:11** | 0.17 |  |  |  |  |
|  |  | **B*51:08** | 0.08 |  |  |  |  |
|  |  | **B*78:01** | 0.08 |  |  |  |  |
|  |  | **B*07:05** | 0.08 |  |  |  |  |
|  |  | **B*09:01** | 0.08 |  |  |  |  |
|  |  | **B*15:09** | 0.08 |  |  |  |  |
|  |  | **B*15:16** | 0.08 |  |  |  |  |
|  |  | **B*15:22** | 0.08 |  |  |  |  |
|  |  | **B*15:48** | 0.08 |  |  |  |  |
|  |  | **B*21:01** | 0.08 |  |  |  |  |
|  |  | **B*33:14** | 0.08 |  |  |  |  |
|  |  | **B*35:05** | 0.08 |  |  |  |  |
|  |  | **B*35:15** | 0.08 |  |  |  |  |
|  |  | **B*36:01** | 0.08 |  |  |  |  |
|  |  | **B*39:03** | 0.08 |  |  |  |  |
|  |  | **B*39:20** | 0.08 |  |  |  |  |
|  |  | **B*40:04** | 0.08 |  |  |  |  |
|  |  | **B*40:12** | 0.08 |  |  |  |  |
|  |  | **B*44:09** | 0.08 |  |  |  |  |
|  |  | **B*44:27** | 0.08 |  |  |  |  |
|  |  | **B*46:01** | 0.08 |  |  |  |  |
|  |  | **B*49:02** | 0.08 |  |  |  |  |
|  |  | **B*51:06** | 0.08 |  |  |  |  |
|  |  | **B*51:07** | 0.08 |  |  |  |  |
|  |  | **B*53:06** | 0.08 |  |  |  |  |
|  |  | **B*54:01** | 0.08 |  |  |  |  |
|  |  | **B*58:02** | 0.08 |  |  |  |  |
|  |  | **B*61:01** | 0.08 |  |  |  |  |
|  |  | **B*65:02** | 0.08 |  |  |  |  |
|  |  | **B*73:01** | 0.08 |  |  |  |  |
|  |  | **B*81:01** | 0.08 |  |  |  |  |
|  |  | **B*82:01** | 0.08 |  |  |  |  |

**Supplementary Table S3.** Complete distribution of inferred four-locus HLA-A~B~C~DRB1 haplotype frequencies in the clinically referred cohort from northeastern Mexico.

| Frequency % | Haplotype |
| --- | --- |
| 1.238 | A*03:01 B*07:02 C*07:02 DRB1*15:01 |
| 1.238 | A*68:03 B*39:05 C*07:02 DRB1*04:07 |
| 1.155 | A*24:02 B*39:06 C*07:02 DRB1*14:06 |
| 1.073 | A*29:02 B*44:03 C*16:01 DRB1*07:01 |
| 0.990 | A*02:01 B*35:17 C*04:01 DRB1*08:02 |
| 0.908 | A*01:01 B*08:01 C*07:01 DRB1*03:01 |
| 0.825 | A*02:01 B*35:12 C*04:01 DRB1*08:02 |
| 0.825 | A*03:01 B*51:01 C*16:01 DRB1*07:01 |
| 0.825 | A*33:01 B*14:02 C*08:02 DRB1*01:02 |
| 0.743 | A*24:02 B*35:01 C*04:01 DRB1*04:04 |
| 0.578 | A*02:01 B*44:03 C*04:01 DRB1*07:01 |
| 0.578 | A*24:02 B*40:05 C*03:04 DRB1*04:04 |
| 0.578 | A*30:02 B*18:01 C*05:01 DRB1*03:01 |
| 0.578 | A*02:06 B*39:05 C*07:02 DRB1*04:07 |
| 0.495 | A*24:02 B*35:12 C*04:01 DRB1*08:02 |
| 0.495 | A*24:02 B*40:02 C*03:06 DRB1*08:02 |
| 0.495 | A*02:01 B*35:12 C*04:01 DRB1*04:07 |
| 0.495 | A*02:01 B*50:01 C*06:02 DRB1*03:01 |
| 0.495 | A*24:02 B*40:05 C*03:04 DRB1*08:02 |
| 0.495 | A*02:01 B*35:01 C*04:01 DRB1*08:02 |
| 0.495 | A*02:01 B*39:05 C*07:02 DRB1*04:07 |
| 0.495 | A*24:02 B*39:05 C*07:02 DRB1*04:07 |
| 0.413 | A*24:02 B*35:43 C*01:02 DRB1*04:07 |
| 0.413 | A*02:01 B*15:15 C*01:02 DRB1*04:03 |
| 0.413 | A*02:01 B*08:01 C*07:01 DRB1*03:01 |
| 0.413 | A*24:02 B*39:06 C*07:02 DRB1*04:04 |
| 0.413 | A*02:01 B*15:15 C*01:02 DRB1*08:02 |
| 0.330 | A*03:01 B*35:03 C*04:01 DRB1*15:01 |
| 0.330 | A*02:01 B*07:02 C*07:02 DRB1*15:01 |
| 0.330 | A*02:05 B*41:01 C*07:01 DRB1*13:05 |
| 0.330 | A*33:01 B*14:02 C*08:02 DRB1*03:01 |
| 0.330 | A*02:01 B*40:02 C*02:02 DRB1*11:01 |
| 0.330 | A*02:01 B*40:02 C*03:04 DRB1*04:04 |
| 0.330 | A*68:02 B*14:02 C*08:02 DRB1*01:02 |
| 0.330 | A*02:01 B*40:02 C*03:06 DRB1*04:11 |
| 0.330 | A*02:06 B*15:30 C*01:02 DRB1*04:03 |
| 0.330 | A*31:01 B*40:02 C*03:04 DRB1*04:11 |
| 0.330 | A*11:01 B*07:02 C*07:02 DRB1*15:01 |
| 0.330 | A*68:01 B*48:01 C*08:01 DRB1*04:04 |
| 0.330 | A*31:01 B*35:12 C*04:01 DRB1*04:07 |
| 0.330 | A*03:01 B*14:02 C*08:02 DRB1*04:05 |
| 0.330 | A*02:01 B*14:02 C*08:02 DRB1*01:02 |
| 0.330 | A*31:01 B*35:01 C*04:01 DRB1*16:02 |
| 0.330 | A*02:01 B*15:01 C*01:02 DRB1*08:02 |
| 0.330 | A*01:01 B*52:01 C*12:02 DRB1*15:02 |
| 0.330 | A*02:01 B*39:02 C*07:02 DRB1*16:02 |
| 0.330 | A*01:01 B*49:01 C*07:01 DRB1*13:02 |
| 0.330 | A*24:02 B*35:02 C*04:01 DRB1*11:04 |
| 0.330 | A*11:01 B*35:01 C*04:01 DRB1*01:03 |
| 0.330 | A*11:01 B*35:01 C*04:01 DRB1*01:01 |
| 0.330 | A*02:06 B*40:02 C*03:04 DRB1*04:07 |
| 0.330 | A*30:02 B*53:01 C*06:02 DRB1*13:02 |
| 0.248 | A*02:01 B*15:03 C*02:10 DRB1*07:01 |
| 0.248 | A*03:01 B*35:03 C*07:02 DRB1*13:02 |
| 0.248 | A*25:01 B*18:01 C*12:03 DRB1*15:01 |
| 0.248 | A*03:01 B*18:01 C*12:03 DRB1*14:07 |
| 0.248 | A*24:02 B*39:06 C*07:02 DRB1*04:07 |
| 0.248 | A*23:01 B*49:01 C*07:01 DRB1*11:04 |
| 0.248 | A*26:01 B*07:02 C*07:02 DRB1*01:01 |
| 0.248 | A*11:01 B*18:01 C*12:03 DRB1*11:04 |
| 0.248 | A*02:01 B*51:01 C*15:02 DRB1*03:01 |
| 0.248 | A*02:06 B*35:01 C*04:01 DRB1*08:02 |
| 0.248 | A*02:01 B*48:01 C*08:01 DRB1*08:02 |
| 0.248 | A*03:01 B*08:01 C*07:01 DRB1*03:01 |
| 0.248 | A*31:01 B*15:01 C*01:02 DRB1*08:02 |
| 0.248 | A*68:02 B*42:02 C*17:01 DRB1*03:02 |
| 0.248 | A*24:02 B*38:01 C*12:03 DRB1*13:01 |
| 0.248 | A*02:01 B*14:01 C*08:02 DRB1*07:01 |
| 0.248 | A*02:01 B*18:01 C*07:01 DRB1*11:04 |
| 0.248 | A*03:01 B*35:01 C*04:01 DRB1*01:01 |
| 0.248 | A*02:01 B*51:01 C*14:02 DRB1*13:01 |
| 0.248 | A*68:03 B*35:01 C*07:02 DRB1*04:07 |
| 0.248 | A*23:01 B*49:01 C*07:01 DRB1*04:05 |
| 0.248 | A*02:01 B*44:03 C*16:01 DRB1*13:01 |
| 0.248 | A*02:01 B*51:01 C*15:02 DRB1*15:01 |
| 0.248 | A*24:02 B*07:02 C*07:02 DRB1*15:01 |
| 0.248 | A*02:06 B*40:02 C*03:06 DRB1*08:02 |
| 0.248 | A*31:01 B*15:30 C*01:02 DRB1*04:04 |
| 0.248 | A*31:01 B*51:02 C*08:01 DRB1*04:07 |
| 0.248 | A*68:01 B*40:02 C*03:05 DRB1*04:04 |
| 0.248 | A*02:06 B*40:02 C*03:04 DRB1*16:02 |
| 0.248 | A*02:01 B*52:01 C*03:03 DRB1*14:06 |
| 0.248 | A*02:01 B*39:06 C*07:02 DRB1*14:06 |
| 0.248 | A*02:01 B*44:02 C*05:01 DRB1*04:03 |
| 0.248 | A*24:02 B*35:01 C*04:01 DRB1*04:07 |
| 0.248 | A*23:01 B*44:03 C*04:01 DRB1*07:01 |
| 0.248 | A*02:01 B*39:02 C*03:04 DRB1*04:04 |
| 0.248 | A*01:01 B*55:01 C*03:03 DRB1*14:54 |
| 0.248 | A*02:01 B*44:02 C*05:01 DRB1*04:01 |
| 0.248 | A*11:01 B*51:01 C*15:02 DRB1*07:01 |
| 0.248 | A*32:01 B*14:01 C*08:02 DRB1*07:01 |
| 0.248 | A*68:01 B*15:15 C*01:02 DRB1*08:02 |
| 0.248 | A*31:01 B*39:01 C*07:02 DRB1*08:02 |
| 0.248 | A*02:06 B*48:01 C*08:01 DRB1*14:02 |
| 0.248 | A*11:01 B*27:05 C*01:02 DRB1*07:01 |
| 0.248 | A*11:01 B*35:03 C*12:03 DRB1*04:08 |
| 0.165 | A*02:06 B*51:01 C*08:01 DRB1*08:02 |
| 0.165 | A*24:02 B*50:01 C*06:02 DRB1*07:01 |
| 0.165 | A*01:01 B*08:01 C*07:01 DRB1*04:01 |
| 0.165 | A*26:01 B*55:01 C*03:03 DRB1*04:04 |
| 0.165 | A*68:03 B*35:12 C*04:01 DRB1*08:02 |
| 0.165 | A*02:01 B*35:01 C*04:01 DRB1*04:07 |
| 0.165 | A*29:02 B*56:01 C*01:02 DRB1*01:01 |
| 0.165 | A*11:01 B*52:01 C*12:02 DRB1*15:02 |
| 0.165 | A*24:02 B*35:01 C*04:01 DRB1*14:54 |
| 0.165 | A*03:01 B*18:01 C*07:01 DRB1*04:03 |
| 0.165 | A*02:01 B*50:01 C*06:02 DRB1*13:01 |
| 0.165 | A*24:02 B*39:08 C*07:02 DRB1*04:07 |
| 0.165 | A*24:02 B*35:01 C*04:01 DRB1*13:02 |
| 0.165 | A*24:02 B*40:02 C*03:06 DRB1*16:02 |
| 0.165 | A*30:01 B*13:02 C*06:02 DRB1*07:01 |
| 0.165 | A*02:06 B*40:02 C*03:03 DRB1*04:11 |
| 0.165 | A*02:01 B*07:02 C*07:02 DRB1*11:01 |
| 0.165 | A*02:06 B*40:08 C*03:04 DRB1*16:02 |
| 0.165 | A*68:01 B*39:05 C*07:02 DRB1*08:02 |
| 0.165 | A*01:01 B*08:01 C*07:01 DRB1*13:02 |
| 0.165 | A*02:01 B*35:03 C*04:01 DRB1*12:01 |
| 0.165 | A*24:02 B*40:02 C*03:04 DRB1*04:07 |
| 0.165 | A*02:05 B*50:02 C*06:02 DRB1*11:03 |
| 0.165 | A*01:01 B*35:02 C*04:01 DRB1*11:04 |
| 0.165 | A*02:01 B*35:01 C*04:01 DRB1*03:01 |
| 0.165 | A*31:01 B*40:01 C*03:04 DRB1*04:04 |
| 0.165 | A*30:01 B*42:01 C*17:01 DRB1*03:02 |
| 0.165 | A*02:01 B*44:02 C*05:01 DRB1*07:01 |
| 0.165 | A*24:02 B*35:01 C*04:01 DRB1*14:02 |
| 0.165 | A*32:01 B*14:02 C*08:02 DRB1*01:02 |
| 0.165 | A*33:01 B*14:02 C*08:02 DRB1*01:01 |
| 0.165 | A*68:01 B*39:05 C*07:02 DRB1*04:11 |
| 0.165 | A*02:01 B*27:05 C*01:02 DRB1*01:01 |
| 0.165 | A*02:06 B*35:01 C*03:04 DRB1*04:04 |
| 0.165 | A*31:01 B*40:08 C*03:04 DRB1*14:06 |
| 0.165 | A*24:02 B*51:01 C*15:09 DRB1*14:06 |
| 0.165 | A*02:01 B*35:08 C*04:01 DRB1*13:02 |
| 0.165 | A*02:01 B*13:02 C*06:02 DRB1*07:01 |
| 0.165 | A*24:02 B*18:01 C*05:01 DRB1*03:01 |
| 0.165 | A*24:02 B*40:02 C*03:08 DRB1*04:11 |
| 0.165 | A*23:01 B*15:03 C*02:10 DRB1*13:01 |
| 0.165 | A*30:01 B*49:01 C*07:01 DRB1*04:05 |
| 0.165 | A*24:02 B*49:01 C*07:01 DRB1*01:02 |
| 0.165 | A*02:01 B*07:02 C*07:02 DRB1*08:02 |
| 0.165 | A*25:01 B*35:01 C*03:03 DRB1*04:11 |
| 0.165 | A*23:01 B*49:01 C*07:01 DRB1*13:04 |
| 0.165 | A*26:01 B*27:05 C*01:02 DRB1*03:01 |
| 0.165 | A*02:01 B*44:03 C*04:01 DRB1*13:01 |
| 0.165 | A*01:01 B*37:01 C*06:02 DRB1*13:01 |
| 0.165 | A*02:01 B*52:01 C*03:03 DRB1*15:01 |
| 0.165 | A*31:01 B*15:30 C*01:02 DRB1*08:02 |
| 0.165 | A*68:01 B*35:01 C*04:01 DRB1*04:07 |
| 0.165 | A*02:01 B*15:01 C*03:03 DRB1*04:04 |
| 0.165 | A*02:01 B*39:01 C*07:02 DRB1*08:02 |
| 0.165 | A*31:01 B*35:17 C*04:01 DRB1*08:02 |
| 0.165 | A*31:01 B*35:01 C*04:01 DRB1*03:01 |
| 0.165 | A*29:02 B*49:01 C*07:01 DRB1*15:03 |
| 0.165 | A*26:01 B*38:01 C*12:03 DRB1*04:02 |
| 0.165 | A*02:01 B*40:02 C*02:02 DRB1*11:04 |
| 0.165 | A*02:01 B*45:01 C*16:01 DRB1*03:02 |
| 0.165 | A*24:02 B*40:02 C*03:05 DRB1*04:07 |
| 0.165 | A*24:02 B*40:05 C*03:04 DRB1*14:02 |
| 0.165 | A*68:01 B*40:02 C*03:04 DRB1*04:07 |
| 0.165 | A*68:01 B*40:05 C*03:04 DRB1*14:02 |
| 0.165 | A*68:05 B*39:08 C*07:02 DRB1*04:07 |
| 0.165 | A*02:01 B*53:01 C*06:02 DRB1*16:02 |
| 0.165 | A*68:01 B*39:06 C*07:02 DRB1*04:04 |
| 0.165 | A*02:01 B*15:30 C*01:02 DRB1*14:06 |
| 0.165 | A*02:01 B*44:02 C*05:01 DRB1*13:01 |
| 0.165 | A*02:01 B*51:01 C*15:09 DRB1*07:01 |
| 0.165 | A*33:01 B*14:02 C*08:02 DRB1*13:02 |
| 0.165 | A*02:01 B*35:01 C*15:09 DRB1*04:04 |
| 0.165 | A*02:01 B*51:01 C*07:01 DRB1*07:01 |
| 0.165 | A*02:01 B*35:14 C*04:01 DRB1*16:02 |
| 0.165 | A*02:05 B*35:01 C*07:02 DRB1*04:07 |
| 0.165 | A*02:01 B*49:01 C*07:01 DRB1*13:02 |
| 0.165 | A*02:06 B*39:05 C*07:02 DRB1*08:02 |
| 0.165 | A*68:02 B*78:01 C*16:01 DRB1*13:01 |
| 0.165 | A*31:01 B*44:02 C*05:01 DRB1*13:01 |
| 0.165 | A*02:20 B*55:01 C*03:03 DRB1*04:07 |
| 0.165 | A*02:01 B*07:02 C*07:02 DRB1*01:03 |
| 0.165 | A*68:05 B*15:01 C*01:02 DRB1*14:06 |
| 0.165 | A*24:02 B*40:02 C*03:06 DRB1*14:06 |
| 0.165 | A*02:01 B*35:01 C*03:05 DRB1*14:02 |
| 0.165 | A*30:02 B*15:03 C*02:10 DRB1*08:04 |
| 0.165 | A*11:01 B*44:03 C*16:01 DRB1*07:01 |
| 0.165 | A*24:02 B*39:06 C*07:02 DRB1*01:01 |
| 0.165 | A*11:01 B*27:05 C*01:02 DRB1*04:02 |
| 0.165 | A*11:01 B*27:05 C*02:02 DRB1*04:04 |
| 0.165 | A*24:02 B*15:35 C*07:02 DRB1*12:02 |
| 0.165 | A*02:01 B*15:03 C*02:10 DRB1*11:02 |
| 0.165 | A*32:01 B*52:01 C*12:02 DRB1*15:02 |
| 0.165 | A*24:25 B*48:01 C*08:01 DRB1*04:11 |
| 0.083 | A*24:02 B*39:06 C*07:02 DRB1*14:02 |
| 0.083 | A*02:06 B*35:12 C*04:01 DRB1*08:02 |
| 0.083 | A*68:05 B*35:12 C*15:02 DRB1*08:02 |
| 0.083 | A*02:05 B*41:01 C*17:01 DRB1*13:02 |
| 0.083 | A*24:02 B*38:01 C*12:03 DRB1*14:01 |
| 0.083 | A*33:01 B*14:02 C*06:02 DRB1*04:04 |
| 0.083 | A*32:01 B*35:03 C*07:02 DRB1*13:01 |
| 0.083 | A*01:01 B*40:06 C*08:01 DRB1*09:01 |
| 0.083 | A*02:06 B*07:02 C*07:02 DRB1*08:02 |
| 0.083 | A*02:01 B*35:03 C*12:03 DRB1*14:01 |
| 0.083 | A*02:01 B*07:02 C*07:01 DRB1*03:01 |
| 0.083 | A*02:01 B*49:01 C*07:02 DRB1*10:01 |
| 0.083 | A*29:02 B*53:01 C*05:01 DRB1*03:01 |
| 0.083 | A*34:02 B*16:01 C*06:02 DRB1*03:01 |
| 0.083 | A*03:01 B*46:01 C*05:01 DRB1*04:04 |
| 0.083 | A*68:01 B*18:01 C*08:01 DRB1*03:01 |
| 0.083 | A*24:02 B*52:01 C*03:03 DRB1*04:01 |
| 0.083 | A*02:01 B*38:01 C*12:03 DRB1*01:01 |
| 0.083 | A*03:01 B*52:01 C*07:02 DRB1*08:03 |
| 0.083 | A*01:02 B*16:01 C*16:01 DRB1*14:54 |
| 0.083 | A*29:02 B*35:01 C*04:01 DRB1*11:04 |
| 0.083 | A*02:01 B*07:02 C*04:01 DRB1*04:02 |
| 0.083 | A*11:01 B*35:08 C*07:02 DRB1*01:01 |
| 0.083 | A*02:01 B*51:01 C*03:04 DRB1*04:07 |
| 0.083 | A*24:23 B*14:01 C*06:02 DRB1*07:01 |
| 0.083 | A*11:01 B*51:01 C*16:02 DRB1*13:02 |
| 0.083 | A*24:02 B*40:02 C*03:04 DRB1*04:04 |
| 0.083 | A*02:05 B*58:01 C*07:01 DRB1*14:06 |
| 0.083 | A*26:01 B*41:01 C*07:01 DRB1*13:03 |
| 0.083 | A*68:07 B*35:01 C*04:01 DRB1*04:07 |
| 0.083 | A*02:01 B*15:01 C*03:03 DRB1*14:06 |
| 0.083 | A*68:07 B*51:07 C*01:02 DRB1*04:03 |
| 0.083 | A*24:02 B*16:01 C*07:01 DRB1*15:01 |
| 0.083 | A*26:01 B*52:01 C*03:03 DRB1*08:02 |
| 0.083 | A*01:02 B*49:01 C*12:03 DRB1*04:05 |
| 0.083 | A*24:02 B*35:08 C*04:01 DRB1*11:04 |
| 0.083 | A*33:01 B*44:03 C*02:02 DRB1*01:02 |
| 0.083 | A*24:25 B*18:01 C*02:02 DRB1*04:11 |
| 0.083 | A*02:01 B*44:03 C*15:02 DRB1*07:01 |
| 0.083 | A*02:05 B*50:01 C*06:02 DRB1*01:03 |
| 0.083 | A*66:01 B*35:28 C*04:01 DRB1*08:02 |
| 0.083 | A*02:01 B*27:05 C*16:02 DRB1*04:02 |
| 0.083 | A*31:01 B*44:03 C*02:02 DRB1*04:04 |
| 0.083 | A*32:01 B*44:02 C*01:02 DRB1*11:01 |
| 0.083 | A*69:01 B*55:01 C*16:04 DRB1*11:04 |
| 0.083 | A*11:01 B*52:01 C*12:02 DRB1*15:01 |
| 0.083 | A*68:01 B*15:03 C*02:10 DRB1*04:07 |
| 0.083 | A*11:01 B*50:01 C*01:02 DRB1*01:01 |
| 0.083 | A*30:02 B*27:05 C*06:02 DRB1*08:06 |
| 0.083 | A*24:02 B*52:01 C*03:03 DRB1*04:11 |
| 0.083 | A*30:01 B*35:03 C*06:02 DRB1*01:01 |
| 0.083 | A*30:02 B*42:01 C*17:01 DRB1*03:02 |
| 0.083 | A*24:01 B*39:05 C*07:02 DRB1*08:02 |
| 0.083 | A*32:01 B*49:01 C*07:01 DRB1*04:02 |
| 0.083 | A*02:01 B*40:05 C*03:04 DRB1*04:04 |
| 0.083 | A*26:03 B*38:01 C*12:03 DRB1*14:54 |
| 0.083 | A*02:01 B*15:01 C*04:01 DRB1*04:04 |
| 0.083 | A*68:05 B*35:20 C*08:01 DRB1*08:01 |
| 0.083 | A*11:01 B*18:01 C*07:01 DRB1*15:01 |
| 0.083 | A*03:01 B*18:01 C*15:02 DRB1*11:04 |
| 0.083 | A*68:02 B*51:01 C*12:03 DRB1*07:01 |
| 0.083 | A*02:01 B*15:03 C*02:10 DRB1*15:01 |
| 0.083 | A*68:01 B*18:01 C*12:03 DRB1*11:04 |
| 0.083 | A*02:01 B*14:02 C*08:02 DRB1*11:01 |
| 0.083 | A*03:01 B*49:01 C*07:01 DRB1*13:03 |
| 0.083 | A*68:01 B*18:01 C*12:03 DRB1*01:01 |
| 0.083 | A*33:01 B*44:03 C*16:01 DRB1*07:01 |
| 0.083 | A*32:01 B*15:01 C*03:04 DRB1*11:01 |
| 0.083 | A*03:01 B*51:01 C*02:02 DRB1*13:01 |
| 0.083 | A*24:02 B*35:01 C*04:01 DRB1*16:02 |
| 0.083 | A*03:01 B*07:02 C*07:02 DRB1*11:04 |
| 0.083 | A*02:06 B*39:03 C*03:04 DRB1*14:02 |
| 0.083 | A*24:02 B*40:06 C*07:02 DRB1*14:02 |
| 0.083 | A*03:01 B*51:01 C*02:02 DRB1*11:01 |
| 0.083 | A*02:01 B*57:01 C*12:03 DRB1*16:01 |
| 0.083 | A*25:01 B*18:01 C*07:01 DRB1*16:01 |
| 0.083 | A*02:01 B*40:02 C*03:03 DRB1*04:07 |
| 0.083 | A*02:01 B*51:02 C*04:01 DRB1*04:11 |
| 0.083 | A*02:06 B*35:02 C*08:01 DRB1*04:03 |
| 0.083 | A*68:05 B*40:08 C*03:04 DRB1*14:02 |
| 0.083 | A*68:02 B*18:01 C*05:01 DRB1*03:01 |
| 0.083 | A*01:01 B*35:16 C*06:02 DRB1*14:06 |
| 0.083 | A*02:01 B*08:01 C*04:01 DRB1*08:01 |
| 0.083 | A*30:02 B*50:01 C*07:02 DRB1*08:01 |
| 0.083 | A*31:01 B*39:05 C*06:02 DRB1*08:01 |
| 0.083 | A*29:02 B*15:01 C*03:06 DRB1*11:01 |
| 0.083 | A*33:01 B*14:02 C*08:02 DRB1*14:01 |
| 0.083 | A*24:02 B*39:05 C*08:02 DRB1*04:04 |
| 0.083 | A*31:01 B*40:02 C*03:02 DRB1*13:02 |
| 0.083 | A*33:01 B*58:01 C*15:02 DRB1*14:24 |
| 0.083 | A*29:02 B*44:03 C*07:01 DRB1*08:01 |
| 0.083 | A*68:03 B*39:05 C*16:01 DRB1*04:07 |
| 0.083 | A*68:01 B*48:01 C*08:01 DRB1*14:06 |
| 0.083 | A*03:01 B*14:02 C*03:02 DRB1*04:01 |
| 0.083 | A*68:02 B*40:02 C*08:02 DRB1*08:02 |
| 0.083 | A*02:06 B*55:01 C*15:02 DRB1*14:01 |
| 0.083 | A*11:01 B*51:01 C*03:03 DRB1*04:11 |
| 0.083 | A*24:02 B*48:01 C*08:01 DRB1*14:02 |
| 0.083 | A*02:01 B*51:01 C*16:02 DRB1*07:01 |
| 0.083 | A*24:02 B*48:01 C*03:06 DRB1*04:04 |
| 0.083 | A*11:01 B*18:01 C*07:01 DRB1*03:01 |
| 0.083 | A*69:01 B*51:01 C*14:02 DRB1*12:02 |
| 0.083 | A*03:01 B*07:02 C*07:02 DRB1*14:54 |
| 0.083 | A*02:01 B*35:04 C*04:01 DRB1*08:02 |
| 0.083 | A*30:02 B*42:02 C*17:01 DRB1*13:02 |
| 0.083 | A*68:01 B*51:01 C*15:02 DRB1*08:02 |
| 0.083 | A*03:01 B*57:01 C*07:01 DRB1*07:01 |
| 0.083 | A*02:01 B*15:15 C*01:02 DRB1*13:02 |
| 0.083 | A*25:01 B*18:01 C*12:03 DRB1*14:02 |
| 0.083 | A*31:01 B*40:02 C*03:05 DRB1*08:02 |
| 0.083 | A*02:01 B*73:01 C*15:05 DRB1*04:05 |
| 0.083 | A*68:01 B*44:03 C*04:01 DRB1*07:01 |
| 0.083 | A*02:01 B*52:01 C*02:02 DRB1*11:01 |
| 0.083 | A*03:01 B*44:05 C*12:02 DRB1*04:04 |
| 0.083 | A*68:01 B*52:01 C*12:02 DRB1*11:04 |
| 0.083 | A*33:03 B*58:01 C*07:02 DRB1*10:01 |
| 0.083 | A*68:05 B*35:01 C*03:02 DRB1*14:02 |
| 0.083 | A*01:01 B*57:01 C*03:04 DRB1*07:01 |
| 0.083 | A*68:05 B*51:01 C*06:02 DRB1*04:11 |
| 0.083 | A*24:02 B*27:05 C*07:02 DRB1*15:01 |
| 0.083 | A*24:02 B*39:05 C*02:02 DRB1*04:11 |
| 0.083 | A*24:02 B*40:02 C*03:06 DRB1*08:04 |
| 0.083 | A*03:01 B*48:01 C*08:02 DRB1*04:03 |
| 0.083 | A*29:02 B*14:02 C*16:01 DRB1*13:04 |
| 0.083 | A*74:01 B*52:01 C*08:02 DRB1*07:01 |
| 0.083 | A*24:02 B*35:12 C*04:01 DRB1*14:02 |
| 0.083 | A*01:01 B*49:01 C*17:01 DRB1*14:06 |
| 0.083 | A*34:02 B*41:02 C*07:01 DRB1*09:01 |
| 0.083 | A*11:01 B*14:01 C*08:02 DRB1*11:01 |
| 0.083 | A*30:02 B*27:05 C*02:02 DRB1*15:01 |
| 0.083 | A*03:01 B*49:01 C*07:01 DRB1*11:02 |
| 0.083 | A*11:01 B*49:01 C*07:01 DRB1*15:01 |
| 0.083 | A*01:01 B*15:01 C*03:03 DRB1*15:01 |
| 0.083 | A*02:35 B*48:01 C*03:04 DRB1*08:02 |
| 0.083 | A*68:05 B*40:05 C*08:01 DRB1*14:02 |
| 0.083 | A*01:01 B*41:02 C*04:01 DRB1*03:01 |
| 0.083 | A*24:02 B*35:05 C*17:03 DRB1*11:04 |
| 0.083 | A*24:02 B*57:01 C*07:01 DRB1*10:01 |
| 0.083 | A*29:01 B*07:02 C*15:05 DRB1*08:02 |
| 0.083 | A*24:02 B*35:12 C*16:01 DRB1*04:11 |
| 0.083 | A*02:01 B*49:01 C*07:01 DRB1*07:01 |
| 0.083 | A*31:01 B*40:02 C*03:06 DRB1*04:05 |
| 0.083 | A*02:06 B*48:01 C*08:01 DRB1*16:02 |
| 0.083 | A*01:01 B*49:01 C*07:01 DRB1*11:01 |
| 0.083 | A*02:01 B*51:01 C*15:02 DRB1*14:06 |
| 0.083 | A*02:01 B*40:02 C*04:01 DRB1*15:01 |
| 0.083 | A*32:01 B*35:17 C*02:02 DRB1*14:06 |
| 0.083 | A*03:01 B*35:01 C*04:04 DRB1*01:01 |
| 0.083 | A*23:01 B*14:01 C*14:02 DRB1*15:03 |
| 0.083 | A*02:06 B*52:01 C*04:01 DRB1*14:06 |
| 0.083 | A*02:06 B*54:01 C*01:02 DRB1*04:03 |
| 0.083 | A*24:02 B*35:04 C*04:01 DRB1*04:03 |
| 0.083 | A*74:01 B*44:02 C*05:01 DRB1*15:03 |
| 0.083 | A*26:01 B*08:01 C*03:04 DRB1*13:04 |
| 0.083 | A*24:02 B*81:01 C*18:01 DRB1*03:01 |
| 0.083 | A*30:02 B*35:15 C*04:01 DRB1*08:02 |
| 0.083 | A*02:05 B*15:01 C*03:03 DRB1*13:01 |
| 0.083 | A*24:02 B*50:01 C*06:02 DRB1*15:01 |
| 0.083 | A*30:04 B*42:01 C*17:01 DRB1*03:02 |
| 0.083 | A*24:02 B*15:01 C*01:02 DRB1*08:02 |
| 0.083 | A*68:01 B*40:05 C*03:04 DRB1*04:04 |
| 0.083 | A*02:01 B*40:27 C*08:01 DRB1*16:02 |
| 0.083 | A*31:01 B*40:02 C*04:01 DRB1*13:01 |
| 0.083 | A*68:01 B*35:14 C*03:04 DRB1*16:02 |
| 0.083 | A*03:01 B*39:06 C*07:02 DRB1*13:01 |
| 0.083 | A*02:06 B*51:01 C*15:09 DRB1*08:02 |
| 0.083 | A*30:01 B*13:02 C*06:02 DRB1*11:01 |
| 0.083 | A*02:01 B*44:02 C*05:01 DRB1*15:01 |
| 0.083 | A*24:02 B*38:01 C*12:03 DRB1*14:54 |
| 0.083 | A*02:01 B*15:01 C*03:04 DRB1*04:03 |
| 0.083 | A*02:01 B*15:30 C*01:02 DRB1*16:02 |
| 0.083 | A*29:02 B*55:01 C*03:03 DRB1*14:54 |
| 0.083 | A*24:02 B*15:30 C*01:02 DRB1*04:04 |
| 0.083 | A*26:01 B*07:02 C*07:02 DRB1*15:01 |
| 0.083 | A*32:01 B*41:01 C*07:01 DRB1*13:15 |
| 0.083 | A*68:05 B*39:05 C*07:02 DRB1*01:01 |
| 0.083 | A*01:01 B*08:01 C*07:01 DRB1*07:01 |
| 0.083 | A*11:01 B*44:02 C*16:02 DRB1*13:01 |
| 0.083 | A*01:01 B*39:01 C*03:02 DRB1*08:04 |
| 0.083 | A*02:01 B*82:01 C*01:02 DRB1*08:02 |
| 0.083 | A*02:01 B*15:01 C*01:02 DRB1*04:11 |
| 0.083 | A*23:01 B*39:06 C*07:02 DRB1*08:02 |
| 0.083 | A*33:01 B*44:02 C*05:01 DRB1*15:01 |
| 0.083 | A*11:01 B*40:02 C*03:04 DRB1*08:07 |
| 0.083 | A*02:06 B*35:08 C*04:01 DRB1*11:04 |
| 0.083 | A*02:06 B*48:01 C*15:09 DRB1*04:07 |
| 0.083 | A*24:02 B*35:01 C*08:03 DRB1*16:02 |
| 0.083 | A*68:01 B*39:01 C*04:01 DRB1*04:04 |
| 0.083 | A*01:01 B*57:01 C*07:01 DRB1*16:01 |
| 0.083 | A*68:02 B*14:02 C*08:02 DRB1*07:01 |
| 0.083 | A*32:01 B*35:02 C*17:01 DRB1*11:01 |
| 0.083 | A*68:02 B*41:01 C*04:01 DRB1*11:04 |
| 0.083 | A*23:01 B*52:01 C*03:03 DRB1*04:10 |
| 0.083 | A*02:05 B*50:01 C*06:02 DRB1*03:01 |
| 0.083 | A*11:01 B*52:01 C*03:04 DRB1*14:06 |
| 0.083 | A*02:01 B*35:01 C*14:02 DRB1*13:01 |
| 0.083 | A*24:02 B*40:02 C*03:05 DRB1*08:02 |
| 0.083 | A*24:02 B*51:02 C*08:01 DRB1*08:02 |
| 0.083 | A*01:02 B*39:06 C*07:02 DRB1*01:02 |
| 0.083 | A*24:02 B*15:03 C*02:10 DRB1*03:01 |
| 0.083 | A*68:01 B*44:05 C*02:02 DRB1*01:01 |
| 0.083 | A*31:01 B*40:02 C*03:04 DRB1*04:07 |
| 0.083 | A*02:05 B*49:01 C*07:01 DRB1*15:01 |
| 0.083 | A*24:03 B*08:01 C*02:02 DRB1*13:01 |
| 0.083 | A*68:01 B*38:01 C*12:03 DRB1*03:01 |
| 0.083 | A*02:01 B*39:06 C*03:03 DRB1*04:07 |
| 0.083 | A*02:05 B*49:01 C*15:05 DRB1*03:02 |
| 0.083 | A*23:01 B*07:02 C*07:01 DRB1*15:01 |
| 0.083 | A*02:01 B*51:01 C*12:03 DRB1*15:01 |
| 0.083 | A*32:01 B*51:01 C*14:02 DRB1*16:01 |
| 0.083 | A*11:01 B*38:01 C*12:03 DRB1*01:01 |
| 0.083 | A*31:01 B*35:01 C*04:01 DRB1*14:06 |
| 0.083 | A*25:01 B*35:01 C*04:01 DRB1*10:01 |
| 0.083 | A*68:02 B*53:01 C*03:03 DRB1*04:03 |
| 0.083 | A*24:02 B*15:01 C*03:04 DRB1*15:01 |
| 0.083 | A*02:01 B*15:01 C*05:01 DRB1*04:07 |
| 0.083 | A*02:05 B*18:01 C*01:02 DRB1*09:01 |
| 0.083 | A*30:02 B*14:02 C*08:02 DRB1*01:02 |
| 0.083 | A*32:01 B*07:02 C*07:02 DRB1*01:03 |
| 0.083 | A*02:01 B*14:02 C*08:02 DRB1*07:01 |
| 0.083 | A*30:01 B*44:03 C*16:01 DRB1*04:05 |
| 0.083 | A*34:02 B*41:02 C*04:01 DRB1*15:03 |
| 0.083 | A*66:01 B*44:03 C*17:03 DRB1*04:04 |
| 0.083 | A*31:01 B*27:05 C*02:02 DRB1*04:11 |
| 0.083 | A*23:01 B*52:01 C*12:02 DRB1*14:02 |
| 0.083 | A*24:02 B*51:01 C*03:06 DRB1*08:01 |
| 0.083 | A*31:01 B*40:02 C*15:02 DRB1*14:02 |
| 0.083 | A*02:06 B*35:17 C*04:01 DRB1*04:07 |
| 0.083 | A*31:01 B*15:48 C*01:02 DRB1*04:07 |
| 0.083 | A*26:01 B*27:05 C*02:02 DRB1*15:01 |
| 0.083 | A*02:01 B*51:01 C*02:02 DRB1*16:01 |
| 0.083 | A*02:01 B*35:16 C*04:01 DRB1*08:02 |
| 0.083 | A*24:02 B*51:02 C*08:01 DRB1*04:03 |
| 0.083 | A*29:02 B*49:01 C*07:01 DRB1*01:01 |
| 0.083 | A*02:01 B*51:01 C*15:02 DRB1*11:01 |
| 0.083 | A*02:01 B*40:08 C*07:02 DRB1*11:01 |
| 0.083 | A*68:03 B*51:01 C*14:02 DRB1*04:25 |
| 0.083 | A*01:01 B*50:01 C*04:01 DRB1*13:05 |
| 0.083 | A*23:01 B*35:01 C*06:02 DRB1*07:01 |
| 0.083 | A*24:02 B*39:01 C*07:02 DRB1*14:06 |
| 0.083 | A*32:01 B*41:02 C*17:03 DRB1*07:01 |
| 0.083 | A*03:01 B*35:43 C*12:03 DRB1*13:01 |
| 0.083 | A*68:03 B*38:01 C*01:02 DRB1*04:07 |
| 0.083 | A*24:02 B*40:11 C*03:05 DRB1*04:04 |
| 0.083 | A*31:01 B*39:01 C*12:03 DRB1*14:54 |
| 0.083 | A*11:01 B*15:17 C*07:01 DRB1*15:01 |
| 0.083 | A*24:02 B*15:15 C*01:02 DRB1*13:01 |
| 0.083 | A*02:01 B*48:01 C*08:03 DRB1*09:01 |
| 0.083 | A*01:01 B*08:01 C*07:02 DRB1*03:01 |
| 0.083 | A*02:01 B*07:02 C*07:02 DRB1*08:01 |
| 0.083 | A*23:01 B*15:17 C*07:01 DRB1*15:01 |
| 0.083 | A*11:01 B*53:01 C*04:01 DRB1*13:02 |
| 0.083 | A*26:01 B*51:01 C*14:02 DRB1*07:01 |
| 0.083 | A*24:02 B*39:06 C*07:02 DRB1*03:01 |
| 0.083 | A*02:01 B*52:01 C*12:02 DRB1*15:02 |
| 0.083 | A*26:01 B*40:01 C*03:04 DRB1*11:03 |
| 0.083 | A*29:02 B*35:03 C*04:01 DRB1*04:03 |
| 0.083 | A*30:01 B*13:02 C*06:02 DRB1*04:11 |
| 0.083 | A*32:01 B*56:01 C*01:02 DRB1*08:01 |
| 0.083 | A*24:02 B*13:02 C*03:04 DRB1*04:11 |
| 0.083 | A*30:01 B*40:02 C*06:02 DRB1*16:02 |
| 0.083 | A*26:01 B*38:01 C*12:03 DRB1*14:54 |
| 0.083 | A*02:01 B*51:08 C*16:02 DRB1*13:01 |
| 0.083 | A*02:01 B*39:06 C*07:02 DRB1*11:01 |
| 0.083 | A*11:01 B*35:28 C*04:01 DRB1*14:06 |
| 0.083 | A*68:20 B*51:01 C*03:03 DRB1*01:01 |
| 0.083 | A*02:06 B*39:08 C*07:02 DRB1*08:02 |
| 0.083 | A*02:01 B*40:02 C*03:04 DRB1*04:07 |
| 0.083 | A*01:01 B*07:02 C*07:02 DRB1*04:04 |
| 0.083 | A*02:01 B*38:01 C*12:03 DRB1*13:01 |
| 0.083 | A*11:01 B*44:02 C*05:01 DRB1*04:05 |
| 0.083 | A*68:01 B*40:02 C*03:04 DRB1*08:02 |
| 0.083 | A*02:01 B*40:08 C*15:02 DRB1*04:03 |
| 0.083 | A*02:06 B*40:02 C*03:06 DRB1*04:04 |
| 0.083 | A*29:02 B*45:01 C*16:01 DRB1*10:01 |
| 0.083 | A*02:01 B*40:01 C*03:04 DRB1*11:02 |
| 0.083 | A*24:07 B*40:02 C*01:02 DRB1*08:02 |
| 0.083 | A*68:03 B*15:15 C*15:02 DRB1*08:02 |
| 0.083 | A*31:01 B*39:06 C*07:02 DRB1*08:02 |
| 0.083 | A*11:01 B*48:01 C*08:03 DRB1*09:01 |
| 0.083 | A*02:01 B*35:08 C*12:03 DRB1*16:01 |
| 0.083 | A*24:02 B*07:02 C*07:02 DRB1*11:02 |
| 0.083 | A*02:01 B*35:17 C*04:01 DRB1*04:04 |
| 0.083 | A*23:01 B*50:02 C*04:01 DRB1*14:54 |
| 0.083 | A*01:01 B*51:01 C*14:02 DRB1*04:01 |
| 0.083 | A*29:02 B*44:03 C*16:01 DRB1*04:04 |
| 0.083 | A*02:01 B*15:30 C*01:02 DRB1*08:02 |
| 0.083 | A*68:02 B*14:02 C*08:02 DRB1*08:04 |
| 0.083 | A*30:01 B*07:02 C*15:05 DRB1*13:02 |
| 0.083 | A*30:02 B*13:02 C*06:02 DRB1*07:01 |
| 0.083 | A*01:01 B*56:01 C*17:01 DRB1*03:01 |
| 0.083 | A*02:01 B*41:01 C*01:02 DRB1*03:01 |
| 0.083 | A*68:05 B*39:01 C*01:02 DRB1*08:02 |
| 0.083 | A*24:02 B*52:01 C*12:02 DRB1*15:02 |
| 0.083 | A*68:03 B*40:02 C*03:04 DRB1*13:02 |
| 0.083 | A*02:01 B*35:17 C*04:01 DRB1*13:01 |
| 0.083 | A*31:01 B*15:30 C*01:02 DRB1*04:03 |
| 0.083 | A*11:01 B*14:02 C*08:02 DRB1*01:02 |
| 0.083 | A*30:01 B*53:01 C*06:02 DRB1*11:02 |
| 0.083 | A*02:06 B*39:02 C*03:04 DRB1*08:02 |
| 0.083 | A*68:01 B*35:01 C*07:02 DRB1*04:07 |
| 0.083 | A*24:02 B*35:16 C*04:01 DRB1*14:02 |
| 0.083 | A*03:01 B*52:01 C*12:02 DRB1*15:02 |
| 0.083 | A*03:01 B*51:01 C*07:02 DRB1*08:03 |
| 0.083 | A*26:01 B*35:03 C*12:03 DRB1*04:03 |
| 0.083 | A*26:01 B*51:01 C*16:01 DRB1*01:01 |
| 0.083 | A*01:01 B*51:01 C*15:02 DRB1*11:01 |
| 0.083 | A*31:01 B*35:01 C*04:01 DRB1*08:02 |
| 0.083 | A*02:01 B*51:01 C*15:09 DRB1*14:06 |
| 0.083 | A*68:01 B*51:01 C*01:02 DRB1*15:01 |
| 0.083 | A*11:01 B*14:01 C*08:02 DRB1*15:01 |
| 0.083 | A*32:01 B*27:02 C*02:02 DRB1*04:02 |
| 0.083 | A*23:01 B*44:03 C*01:02 DRB1*07:01 |
| 0.083 | A*24:02 B*14:02 C*06:02 DRB1*01:02 |
| 0.083 | A*23:01 B*49:01 C*07:01 DRB1*13:01 |
| 0.083 | A*02:01 B*35:12 C*04:01 DRB1*07:01 |
| 0.083 | A*02:01 B*57:01 C*06:02 DRB1*14:06 |
| 0.083 | A*24:02 B*40:02 C*03:05 DRB1*13:02 |
| 0.083 | A*31:01 B*15:15 C*01:02 DRB1*04:07 |
| 0.083 | A*02:01 B*40:05 C*03:04 DRB1*08:02 |
| 0.083 | A*03:01 B*15:16 C*14:02 DRB1*14:06 |
| 0.083 | A*02:01 B*48:01 C*08:03 DRB1*01:01 |
| 0.083 | A*02:01 B*52:01 C*03:03 DRB1*16:02 |
| 0.083 | A*02:01 B*07:02 C*07:02 DRB1*07:01 |
| 0.083 | A*23:01 B*49:01 C*04:01 DRB1*03:01 |
| 0.083 | A*24:02 B*44:03 C*16:01 DRB1*11:04 |
| 0.083 | A*30:02 B*35:02 C*07:01 DRB1*07:01 |
| 0.083 | A*02:01 B*52:01 C*03:03 DRB1*10:01 |
| 0.083 | A*31:01 B*44:03 C*16:01 DRB1*13:02 |
| 0.083 | A*32:01 B*35:01 C*04:01 DRB1*07:01 |
| 0.083 | A*02:06 B*51:01 C*07:02 DRB1*08:02 |
| 0.083 | A*68:01 B*40:27 C*03:04 DRB1*04:08 |
| 0.083 | A*01:01 B*57:01 C*12:03 DRB1*07:01 |
| 0.083 | A*25:01 B*18:01 C*07:01 DRB1*14:54 |
| 0.083 | A*11:01 B*51:02 C*08:01 DRB1*08:02 |
| 0.083 | A*02:01 B*51:01 C*04:01 DRB1*16:02 |
| 0.083 | A*02:06 B*40:08 C*03:04 DRB1*08:02 |
| 0.083 | A*02:01 B*27:05 C*02:02 DRB1*13:01 |
| 0.083 | A*24:02 B*15:01 C*01:02 DRB1*04:07 |
| 0.083 | A*24:25 B*35:17 C*04:01 DRB1*08:02 |
| 0.083 | A*66:01 B*58:02 C*06:02 DRB1*15:03 |
| 0.083 | A*03:01 B*07:02 C*07:02 DRB1*08:01 |
| 0.083 | A*68:01 B*15:01 C*01:02 DRB1*16:02 |
| 0.083 | A*68:02 B*08:01 C*15:02 DRB1*08:02 |
| 0.083 | A*01:01 B*35:14 C*04:01 DRB1*16:02 |
| 0.083 | A*24:02 B*38:01 C*12:03 DRB1*13:02 |
| 0.083 | A*24:02 B*57:01 C*04:01 DRB1*07:01 |
| 0.083 | A*24:07 B*15:01 C*01:02 DRB1*04:04 |
| 0.083 | A*68:03 B*40:05 C*03:04 DRB1*08:02 |
| 0.083 | A*66:01 B*18:01 C*17:01 DRB1*04:04 |
| 0.083 | A*68:02 B*41:02 C*05:01 DRB1*13:04 |
| 0.083 | A*68:01 B*15:03 C*12:03 DRB1*08:04 |
| 0.083 | A*02:01 B*45:01 C*16:01 DRB1*04:04 |
| 0.083 | A*03:01 B*14:02 C*04:01 DRB1*01:02 |
| 0.083 | A*33:01 B*35:01 C*08:32 DRB1*11:01 |
| 0.083 | A*68:01 B*35:03 C*08:01 DRB1*04:07 |
| 0.083 | A*68:01 B*48:01 C*04:01 DRB1*16:01 |
| 0.083 | A*32:01 B*49:01 C*02:02 DRB1*03:01 |
| 0.083 | A*11:01 B*44:05 C*02:02 DRB1*01:01 |
| 0.083 | A*30:02 B*18:01 C*05:01 DRB1*04:04 |
| 0.083 | A*02:01 B*51:01 C*07:01 DRB1*09:01 |
| 0.083 | A*25:01 B*51:01 C*03:03 DRB1*13:01 |
| 0.083 | A*02:01 B*39:05 C*03:03 DRB1*04:03 |
| 0.083 | A*31:01 B*52:01 C*07:02 DRB1*14:02 |
| 0.083 | A*02:01 B*15:09 C*07:04 DRB1*11:01 |
| 0.083 | A*68:01 B*42:02 C*17:01 DRB1*08:02 |
| 0.083 | A*74:01 B*35:01 C*04:01 DRB1*13:01 |
| 0.083 | A*01:01 B*41:01 C*17:01 DRB1*10:01 |
| 0.083 | A*11:01 B*55:01 C*03:03 DRB1*07:01 |
| 0.083 | A*25:01 B*51:01 C*15:02 DRB1*13:01 |
| 0.083 | A*68:02 B*39:05 C*07:02 DRB1*04:07 |
| 0.083 | A*24:02 B*35:01 C*04:01 DRB1*04:05 |
| 0.083 | A*68:05 B*15:01 C*03:04 DRB1*16:02 |
| 0.083 | A*02:02 B*53:01 C*04:01 DRB1*13:03 |
| 0.083 | A*02:06 B*48:01 C*08:01 DRB1*04:05 |
| 0.083 | A*03:01 B*15:01 C*03:04 DRB1*16:02 |
| 0.083 | A*24:07 B*39:05 C*07:02 DRB1*13:01 |
| 0.083 | A*68:03 B*38:01 C*12:03 DRB1*16:02 |
| 0.083 | A*01:01 B*49:01 C*07:01 DRB1*15:01 |
| 0.083 | A*32:01 B*07:02 C*07:01 DRB1*03:01 |
| 0.083 | A*29:02 B*44:03 C*16:01 DRB1*03:01 |
| 0.083 | A*31:02 B*52:01 C*03:03 DRB1*14:02 |
| 0.083 | A*02:02 B*08:01 C*03:04 DRB1*13:04 |
| 0.083 | A*68:03 B*57:01 C*07:01 DRB1*15:01 |
| 0.083 | A*02:01 B*44:02 C*05:01 DRB1*09:01 |
| 0.083 | A*02:01 B*51:01 C*14:02 DRB1*09:06 |
| 0.083 | A*26:01 B*35:01 C*04:01 DRB1*13:01 |
| 0.083 | A*02:20 B*38:01 C*12:03 DRB1*13:01 |
| 0.083 | A*24:07 B*40:27 C*04:01 DRB1*04:04 |
| 0.083 | A*68:03 B*35:02 C*03:04 DRB1*11:04 |
| 0.083 | A*03:01 B*35:03 C*04:01 DRB1*11:01 |
| 0.083 | A*02:11 B*07:05 C*15:02 DRB1*10:01 |
| 0.083 | A*29:01 B*40:06 C*15:05 DRB1*15:01 |
| 0.083 | A*02:01 B*18:01 C*07:01 DRB1*08:01 |
| 0.083 | A*68:02 B*15:10 C*07:35 DRB1*15:03 |
| 0.083 | A*68:02 B*49:01 C*03:04 DRB1*11:01 |
| 0.083 | A*23:01 B*37:01 C*03:03 DRB1*07:01 |
| 0.083 | A*24:02 B*55:01 C*06:02 DRB1*16:01 |
| 0.083 | A*02:01 B*55:01 C*03:03 DRB1*03:01 |
| 0.083 | A*24:02 B*08:01 C*07:02 DRB1*16:01 |
| 0.083 | A*68:05 B*35:17 C*04:01 DRB1*14:02 |
| 0.083 | A*36:01 B*53:01 C*04:01 DRB1*07:01 |
| 0.083 | A*33:01 B*15:15 C*01:02 DRB1*04:07 |
| 0.083 | A*01:01 B*37:01 C*06:02 DRB1*04:04 |
| 0.083 | A*24:02 B*35:17 C*04:01 DRB1*14:06 |
| 0.083 | A*29:01 B*35:08 C*04:01 DRB1*04:03 |
| 0.083 | A*02:01 B*40:02 C*03:04 DRB1*04:11 |
| 0.083 | A*24:02 B*44:05 C*02:02 DRB1*04:05 |
| 0.083 | A*32:01 B*50:01 C*06:02 DRB1*04:05 |
| 0.083 | A*30:02 B*48:01 C*08:03 DRB1*13:01 |
| 0.083 | A*31:01 B*35:01 C*04:01 DRB1*04:04 |
| 0.083 | A*31:01 B*51:01 C*12:03 DRB1*11:04 |
| 0.083 | A*33:01 B*44:03 C*04:01 DRB1*07:01 |
| 0.083 | A*74:01 B*49:01 C*07:01 DRB1*15:03 |
| 0.083 | A*01:01 B*58:01 C*07:01 DRB1*07:01 |
| 0.083 | A*11:01 B*38:01 C*05:01 DRB1*03:01 |
| 0.083 | A*31:01 B*18:01 C*12:03 DRB1*13:02 |
| 0.083 | A*30:01 B*42:01 C*17:01 DRB1*09:01 |
| 0.083 | A*23:01 B*44:03 C*15:02 DRB1*08:04 |
| 0.083 | A*33:01 B*35:02 C*04:01 DRB1*11:04 |
| 0.083 | A*68:01 B*48:01 C*08:01 DRB1*08:02 |
| 0.083 | A*02:06 B*14:02 C*08:02 DRB1*13:03 |
| 0.083 | A*31:01 B*48:01 C*08:01 DRB1*04:04 |
| 0.083 | A*02:09 B*35:08 C*04:01 DRB1*04:03 |
| 0.083 | A*31:02 B*40:02 C*15:02 DRB1*04:07 |
| 0.083 | A*68:12 B*51:01 C*03:04 DRB1*16:02 |
| 0.083 | A*01:01 B*27:02 C*04:01 DRB1*11:01 |
| 0.083 | A*26:01 B*35:03 C*02:02 DRB1*11:01 |
| 0.083 | A*23:01 B*53:01 C*04:82 DRB1*08:04 |
| 0.083 | A*68:05 B*35:16 C*04:01 DRB1*08:02 |
| 0.083 | A*33:01 B*15:15 C*01:02 DRB1*04:10 |
| 0.083 | A*23:01 B*41:01 C*07:01 DRB1*08:04 |
| 0.083 | A*31:01 B*51:02 C*08:01 DRB1*08:02 |
| 0.083 | A*68:01 B*35:28 C*04:01 DRB1*08:02 |
| 0.083 | A*31:01 B*35:43 C*01:02 DRB1*04:07 |
| 0.083 | A*68:03 B*35:01 C*03:04 DRB1*04:07 |
| 0.083 | A*02:05 B*15:22 C*12:03 DRB1*14:54 |
| 0.083 | A*68:05 B*39:02 C*03:04 DRB1*04:03 |
| 0.083 | A*31:01 B*07:02 C*07:02 DRB1*08:01 |
| 0.083 | A*32:01 B*51:08 C*16:02 DRB1*15:01 |
| 0.083 | A*02:01 B*49:01 C*07:01 DRB1*11:02 |
| 0.083 | A*02:05 B*27:05 C*04:01 DRB1*03:01 |
| 0.083 | A*69:01 B*55:01 C*01:02 DRB1*11:02 |
| 0.083 | A*02:01 B*51:01 C*15:02 DRB1*14:54 |
| 0.083 | A*29:02 B*18:01 C*16:01 DRB1*03:01 |
| 0.083 | A*30:01 B*13:02 C*06:02 DRB1*14:02 |
| 0.083 | A*68:01 B*48:01 C*08:03 DRB1*16:02 |
| 0.083 | A*02:01 B*44:02 C*04:01 DRB1*11:04 |
| 0.083 | A*68:03 B*35:12 C*07:04 DRB1*04:03 |
| 0.083 | A*31:01 B*40:02 C*08:01 DRB1*03:02 |
| 0.083 | A*68:01 B*35:12 C*04:01 DRB1*14:06 |
| 0.083 | A*33:01 B*48:01 C*08:01 DRB1*04:04 |
| 0.083 | A*32:01 B*35:03 C*04:01 DRB1*15:01 |
| 0.083 | A*02:02 B*35:12 C*17:01 DRB1*08:02 |
| 0.083 | A*31:01 B*41:01 C*04:01 DRB1*07:01 |
| 0.083 | A*25:01 B*51:01 C*03:03 DRB1*07:01 |
| 0.083 | A*24:02 B*39:02 C*07:02 DRB1*04:07 |
| 0.083 | A*01:01 B*44:02 C*05:01 DRB1*15:01 |
| 0.083 | A*03:01 B*49:01 C*07:01 DRB1*15:01 |
| 0.083 | A*02:01 B*39:06 C*07:02 DRB1*16:02 |
| 0.083 | A*01:01 B*15:15 C*01:02 DRB1*08:02 |
| 0.083 | A*02:01 B*51:01 C*02:02 DRB1*15:01 |
| 0.083 | A*68:03 B*35:28 C*04:01 DRB1*08:02 |
| 0.083 | A*03:01 B*13:02 C*06:02 DRB1*07:01 |
| 0.083 | A*23:01 B*35:08 C*04:01 DRB1*13:05 |
| 0.083 | A*24:02 B*35:03 C*04:01 DRB1*01:03 |
| 0.083 | A*02:01 B*51:01 C*05:01 DRB1*14:24 |
| 0.083 | A*02:02 B*18:01 C*14:02 DRB1*08:01 |
| 0.083 | A*03:01 B*35:02 C*04:01 DRB1*11:04 |
| 0.083 | A*30:02 B*58:01 C*03:02 DRB1*09:01 |
| 0.083 | A*02:01 B*37:01 C*06:02 DRB1*10:01 |
| 0.083 | A*26:01 B*38:01 C*12:03 DRB1*13:01 |
| 0.083 | A*26:01 B*51:01 C*15:02 DRB1*03:01 |
| 0.083 | A*24:02 B*37:01 C*06:02 DRB1*11:01 |
| 0.083 | A*24:02 B*15:17 C*07:01 DRB1*13:02 |
| 0.083 | A*01:17 B*48:01 C*08:01 DRB1*15:02 |
| 0.083 | A*68:03 B*52:01 C*12:02 DRB1*04:07 |
| 0.083 | A*23:01 B*49:01 C*07:01 DRB1*09:01 |
| 0.083 | A*30:02 B*55:01 C*03:03 DRB1*15:01 |
| 0.083 | A*02:01 B*44:09 C*05:01 DRB1*01:01 |
| 0.083 | A*02:01 B*51:01 C*14:02 DRB1*04:03 |
| 0.083 | A*68:03 B*39:08 C*07:17 DRB1*08:02 |
| 0.083 | A*02:01 B*27:05 C*02:02 DRB1*14:02 |
| 0.083 | A*01:01 B*58:01 C*07:18 DRB1*07:01 |
| 0.083 | A*29:02 B*15:03 C*16:01 DRB1*15:01 |
| 0.083 | A*24:02 B*39:05 C*07:02 DRB1*08:02 |
| 0.083 | A*26:01 B*44:03 C*04:01 DRB1*07:01 |
| 0.083 | A*31:09 B*39:06 C*07:02 DRB1*04:04 |
| 0.083 | A*02:06 B*15:15 C*01:02 DRB1*04:10 |
| 0.083 | A*03:02 B*51:01 C*15:02 DRB1*04:02 |
| 0.083 | A*02:01 B*35:43 C*01:02 DRB1*01:01 |
| 0.083 | A*32:01 B*39:01 C*12:03 DRB1*04:07 |
| 0.083 | A*02:06 B*27:05 C*04:01 DRB1*14:02 |
| 0.083 | A*24:02 B*35:08 C*02:02 DRB1*14:54 |
| 0.083 | A*11:01 B*50:01 C*06:02 DRB1*08:04 |
| 0.083 | A*02:01 B*39:02 C*07:02 DRB1*04:07 |
| 0.083 | A*23:01 B*35:01 C*04:01 DRB1*11:04 |
| 0.083 | A*02:01 B*07:02 C*03:03 DRB1*01:01 |
| 0.083 | A*11:01 B*51:01 C*07:02 DRB1*09:01 |
| 0.083 | A*32:01 B*35:08 C*04:01 DRB1*11:01 |
| 0.083 | A*02:01 B*35:14 C*04:01 DRB1*08:01 |
| 0.083 | A*02:01 B*39:06 C*07:02 DRB1*08:02 |
| 0.083 | A*26:01 B*51:01 C*02:02 DRB1*14:02 |
| 0.083 | A*03:01 B*45:01 C*06:02 DRB1*13:02 |
| 0.083 | A*24:02 B*48:01 C*08:01 DRB1*04:04 |
| 0.083 | A*24:03 B*35:01 C*07:02 DRB1*08:02 |
| 0.083 | A*24:03 B*52:01 C*03:03 DRB1*14:06 |
| 0.083 | A*31:01 B*35:01 C*04:04 DRB1*08:02 |
| 0.083 | A*29:02 B*44:03 C*16:01 DRB1*01:01 |
| 0.083 | A*68:05 B*35:20 C*04:01 DRB1*04:10 |
| 0.083 | A*32:01 B*53:01 C*04:01 DRB1*03:01 |
| 0.083 | A*68:05 B*40:02 C*03:05 DRB1*04:07 |
| 0.083 | A*02:01 B*57:01 C*07:01 DRB1*08:02 |
| 0.083 | A*03:01 B*39:05 C*07:02 DRB1*08:02 |
| 0.083 | A*30:01 B*39:06 C*03:05 DRB1*14:06 |
| 0.083 | A*02:01 B*15:03 C*02:10 DRB1*08:03 |
| 0.083 | A*29:02 B*44:27 C*07:04 DRB1*13:01 |
| 0.083 | A*68:01 B*44:02 C*07:04 DRB1*16:01 |
| 0.083 | A*02:01 B*57:01 C*07:04 DRB1*13:01 |
| 0.083 | A*68:01 B*44:02 C*06:02 DRB1*11:01 |
| 0.083 | A*03:01 B*38:01 C*02:10 DRB1*03:01 |
| 0.083 | A*32:01 B*15:03 C*12:03 DRB1*07:01 |
| 0.083 | A*68:03 B*48:01 C*04:01 DRB1*14:02 |
| 0.083 | A*29:02 B*44:03 C*16:01 DRB1*15:01 |
| 0.083 | A*02:01 B*49:01 C*07:01 DRB1*09:01 |
| 0.083 | A*26:01 B*15:17 C*07:01 DRB1*04:05 |
| 0.083 | A*33:01 B*14:02 C*08:02 DRB1*04:11 |
| 0.083 | A*66:01 B*44:03 C*16:02 DRB1*04:02 |
| 0.083 | A*03:01 B*08:01 C*07:01 DRB1*13:01 |
| 0.083 | A*68:05 B*07:02 C*07:02 DRB1*16:01 |
| 0.083 | A*68:02 B*35:01 C*04:01 DRB1*16:01 |
| 0.083 | A*02:01 B*44:02 C*05:01 DRB1*15:02 |
| 0.083 | A*24:15 B*15:01 C*01:02 DRB1*15:02 |
| 0.083 | A*02:01 B*48:01 C*04:01 DRB1*08:02 |
| 0.083 | A*02:01 B*35:43 C*01:02 DRB1*07:01 |
| 0.083 | A*03:01 B*07:02 C*07:02 DRB1*04:11 |
| 0.083 | A*01:01 B*35:08 C*04:01 DRB1*11:04 |
| 0.083 | A*68:01 B*40:11 C*03:04 DRB1*04:07 |
| 0.083 | A*02:01 B*40:08 C*03:04 DRB1*14:06 |
| 0.083 | A*01:02 B*49:01 C*07:01 DRB1*13:02 |
| 0.083 | A*24:02 B*35:17 C*04:01 DRB1*08:04 |
| 0.083 | A*26:01 B*51:01 C*08:01 DRB1*04:07 |
| 0.083 | A*26:01 B*40:02 C*02:02 DRB1*01:02 |
| 0.083 | A*30:01 B*42:01 C*17:01 DRB1*11:04 |
| 0.083 | A*02:05 B*58:01 C*07:18 DRB1*13:02 |
| 0.083 | A*11:01 B*49:01 C*07:01 DRB1*04:05 |
| 0.083 | A*02:06 B*39:06 C*07:02 DRB1*14:06 |
| 0.083 | A*01:01 B*37:01 C*06:02 DRB1*07:01 |
| 0.083 | A*02:01 B*38:01 C*12:03 DRB1*11:01 |
| 0.083 | A*24:02 B*51:02 C*14:02 DRB1*12:02 |
| 0.083 | A*01:01 B*35:01 C*04:01 DRB1*13:02 |
| 0.083 | A*32:01 B*35:01 C*03:04 DRB1*07:01 |
| 0.083 | A*68:01 B*50:01 C*06:02 DRB1*08:02 |
| 0.083 | A*03:01 B*39:20 C*12:03 DRB1*08:04 |
| 0.083 | A*30:02 B*15:01 C*05:01 DRB1*11:01 |
| 0.083 | A*02:06 B*48:01 C*08:03 DRB1*08:02 |
| 0.083 | A*25:01 B*58:01 C*03:03 DRB1*13:01 |
| 0.083 | A*80:01 B*15:01 C*03:02 DRB1*03:01 |
| 0.083 | A*02:06 B*40:02 C*03:04 DRB1*04:03 |
| 0.083 | A*02:06 B*35:01 C*04:01 DRB1*04:10 |
| 0.083 | A*30:02 B*53:06 C*15:02 DRB1*04:03 |
| 0.083 | A*31:01 B*41:01 C*17:01 DRB1*08:04 |
| 0.083 | A*24:02 B*39:08 C*07:02 DRB1*04:11 |
| 0.083 | A*24:02 B*52:01 C*03:03 DRB1*03:01 |
| 0.083 | A*24:33 B*51:06 C*12:04 DRB1*07:01 |
| 0.083 | A*02:06 B*15:10 C*04:01 DRB1*13:01 |
| 0.083 | A*68:15 B*52:01 C*03:04 DRB1*08:02 |
| 0.083 | A*02:22 B*40:04 C*03:04 DRB1*04:07 |
| 0.083 | A*24:02 B*35:08 C*04:01 DRB1*11:01 |
| 0.083 | A*02:01 B*51:02 C*08:01 DRB1*04:07 |
| 0.083 | A*31:01 B*44:03 C*16:01 DRB1*04:03 |
| 0.083 | A*02:01 B*40:02 C*03:03 DRB1*13:01 |
| 0.083 | A*02:05 B*52:01 C*03:04 DRB1*09:01 |
| 0.083 | A*68:01 B*35:01 C*04:01 DRB1*01:01 |

**Supplementary Table S4.** Reference datasets used for pairwise FST comparisons with the northeastern Mexican cohort.

| Reference dataset | Abbreviation | Sample size used for analysis (n) | AFND source |
| --- | --- | --- | --- |
| Canada Ojibwa NA-DHS_4 (G) | CAN Oji | 15 | [Canada Ojibwa NA-DHS_4 (G)](https://www.allelefrequencies.net/pop6001c.asp?pop_name=Canada%20Ojibwa%20NA-DHS_4%20(G)) |
| Canada Chipewyan NA-DHS_2 (G) | CAN Cph | 25 | [Canada Chipewyan NA-DHS_2 (G)](https://www.allelefrequencies.net/pop6001c.asp?pop_name=Canada%20Chipewyan%20NA-DHS_2%20(G)) |
| United States of America San Diego | USA SD | 310 | [USA San Diego](https://www.allelefrequencies.net/pop6001c.asp?pop_name=USA%20San%20Diego) |
| Mexico Mixe NA-DHS_6 (G) + Mexico Mixtec NA-DHS_5 (G) | MX Mix | 40 | [Mexico Mixe NA-DHS_6 (G)](https://www.allelefrequencies.net/pop6001c.asp?pop_name=Mexico%20Mixe%20NA-DHS_6%20(G)); [Mexico Mixtec NA-DHS_5 (G)](https://www.allelefrequencies.net/pop6001c.asp?pop_name=Mexico%20Mixtec%20NA-DHS_5%20(G)) |
| Mexico Chichen Itza Maya | MX CI-M | 47 | [Mexico Chichen Itza Maya (prehispanic)](https://www.allelefrequencies.net/pop6001c.asp?pop_name=Mexico%20Chichen%20Itza%20Maya%20(prehispanic)) |
| Mexico Tixcacaltuyub Maya | MX Tic-M | 67 | [Mexico Tixcacaltuyub Maya](https://www.allelefrequencies.net/pop6001c.asp?pop_name=Mexico%20Tixcacaltuyub%20Maya) |
| Mexico Chiapas Lacandon Mayans | MX ChiL-M | 218 | [Mexico Chiapas Lacandon Mayans](https://www.allelefrequencies.net/pop6001c.asp?pop_name=Mexico%20Chiapas%20Lacandon%20Mayans) |
| Nicaragua Mestizo (G) | NIC Mest | 154 | [Nicaragua Mestizo (G)](https://www.allelefrequencies.net/pop6001c.asp?pop_name=Nicaragua%20Mestizo%20(G)) |
| Costa Rica Central Valley Mestizo (G) | CR CV Mest | 221 | [Costa Rica Central Valley Mestizo (G)](https://www.allelefrequencies.net/pop6001c.asp?pop_name=Costa%20Rica%20Central%20Valley%20Mestizo%20%20(G)) |
| Costa Rica Guanacaste Mestizo (G) | CR Gte Mest | 110 | [Costa Rica Guanacaste Mestizo (G)](https://www.allelefrequencies.net/pop6001c.asp?pop_name=Costa%20Rica%20Guanacaste%20Mestizo%20(G)) |
| Costa Rica Amerindians (G) | CR Ame | 119 | [Costa Rica Amerindians (G)](https://www.allelefrequencies.net/pop6001c.asp?pop_name=Costa%20Rica%20Amerindians%20(G)) |
| Costa Rica African -Caribbean (G) | CR AF-Car | 102 | [Costa Rica African-Caribbean (G)](https://www.allelefrequencies.net/pop6001c.asp?pop_name=Costa%20Rica%20African%20-Caribbean%20(G)) |
| Colombia Bogotá Cord Blood | COL Bog-CB | 1,463 | [Colombia Bogotá Cord Blood](https://www.allelefrequencies.net/pop6001c.asp?pop_name=Colombia%20Bogot%C3%A1%20Cord%20Blood) |
| Colombia North Chimila Amerindians | COL NC-Ame | 47 | [Colombia North Chimila Amerindians](https://www.allelefrequencies.net/pop6001c.asp?pop_name=Colombia%20North%20Chimila%20Amerindians) |
| Colombia North Wiwa El Encanto | COL N-EE | 52 | [Colombia North Wiwa El Encanto](https://www.allelefrequencies.net/pop6001c.asp?pop_name=Colombia%20North%20Wiwa%20El%20Encanto) |
| Bolivia/Chile Aymara NA-DHS_13 (G) + Bolivia/Peru Quechua NA-DHS_12 (G) | BOL | 41 | [Bolivia/Chile Aymara NA-DHS_13 (G)](https://www.allelefrequencies.net/pop6001c.asp?pop_name=Bolivia/Chile%20Aymara%20NA-DHS_13%20(G)); [Bolivia/Peru Quechua NA-DHS_12 (G)](https://www.allelefrequencies.net/pop6001c.asp?pop_name=Bolivia/Peru%20Quechua%20NA-DHS_12%20(G)) |
| Brazil Puyanawa | BR Pyn | 150 | [Brazil Puyanawa](https://www.allelefrequencies.net/pop6001c.asp?pop_name=Brazil%20%20Puyanawa) |
| Brazil Barra Mansa Rio State Caucasian | BR BM-RJCau | 369 | [Brazil Barra Mansa Rio State Caucasian](https://www.allelefrequencies.net/pop6001c.asp?pop_name=Brazil%20Barra%20Mansa%20Rio%20State%20Caucasian) |
| Brazil Barra Mansa Rio State Black | BR BM-RJBlk | 70 | [Brazil Barra Mansa Rio State Black](https://www.allelefrequencies.net/pop6001c.asp?pop_name=Brazil%20Barra%20Mansa%20Rio%20State%20Black) |
| Brazil Rio de Janeiro Black | BR RJBlk | 47 | [Brazil Rio de Janeiro Black](https://www.allelefrequencies.net/pop6001c.asp?pop_name=Brazil%20Rio%20de%20Janeiro%20Black) |
| Brazil Rio de Janeiro Caucasian | BR-RJCau | 349 | [Brazil Rio de Janeiro Caucasian](https://www.allelefrequencies.net/pop6001c.asp?pop_name=Brazil%20Rio%20de%20Janeiro%20Caucasian) |
| Brazil Rio de Janeiro Parda | BR-RJ-P | 108 | [Brazil Rio de Janeiro Parda](https://www.allelefrequencies.net/pop6001c.asp?pop_name=Brazil%20Rio%20de%20Janeiro%20Parda) |
| *Note: Sample size represents the number of individuals with complete HLA-A, -B, -C, and -DRB1 genotypes included in each comparison. Individual-level genotype data were obtained from the Allele Frequency Net Database (AFND). Four-locus HLA-A~B~C~DRB1 haplotype frequencies were inferred from these genotype data using the EM algorithm implemented in Arlequin v3.5.2.2. For composite datasets, individual-level genotypes from the corresponding AFND populations were combined before haplotype estimation and FST analysis.* | | | |
